# 4-Thiazolidinone coumarin derivatives as two-component NS2B/NS3 DENV flavivirus serine protease inhibitors: synthesis, molecular docking, biological evaluation and structure–activity relationship studies

**DOI:** 10.1186/s13065-018-0435-0

**Published:** 2018-06-12

**Authors:** Samina Khan Yusufzai, Hasnah Osman, Mohammad Shaheen Khan, Basma M. Abd Razik, Mohammed Oday Ezzat, Suriyati Mohamad, Othman Sulaiman, Jualang Azlan Gansau, Thaigarajan Parumasivam

**Affiliations:** 1 School of Chemical Sciences, Universiti Sains Malaysia, 11800 Penang, Malaysia; 2 School of Industrial Technology, Universiti Sains Malaysia, 11800 Penang, Malaysia; 3 Industrial Chemistry Programme, Faculty of Science and Natural Resources, Universiti Malaysia Sabah, 88400 Kota Kinabalu, Sabah Malaysia; 4 College of Pharmacy, Al-Mustansiriyah University, Baghdad, 10001 Iraq; 5 College of Education for Women, University of Anbar, Ramadi, Anbar 31001 Iraq; 6 School of Biological Sciences, Universiti Sains Malaysia, 11800 Penang, Malaysia; 7 Biotechnology Programme, Faculty of Science and Natural Resources, Universiti Malaysia Sabah, 88400 Kota Kinabalu, Sabah Malaysia; 8 School of Pharmaceutical Sciences, Universiti Sains Malaysia, 11800 Penang, Malaysia

**Keywords:** Molecular docking, Anti-bacterial, Anti-tuberculosis, Anti-viral, Anti-dengue, Coumarin thiazolidinone

## Abstract

A series of novel 4-thiazolidinone inhibitors **SKYa**–**SKYg**, containing coumarin as a core structure were synthesized via facile and efficient method. The structures of the synthesized compounds were established by extensive spectroscopic studies (FT IR, 1D NMR, 2D NMR, LC–MS) and elemental analysis. All the synthesized hybrids were further evaluated for their potential as anti-tubercular agents against *Mycobacterium tuberculosis* H37Rv ATCC 25618, and anti-bacterial agents against *Escherichia coli, Enterobacter aerogenes, Salmonella typhi, Streptococcus pneumoniae* and *Staphylococcus aureus*. Interestingly, the hybrids displayed potent bioactivity. However, compounds **SKYc**, **SKYd**, and **SKYe** appeared to be more effective against the tested bacterial strains, among which compound **SKYb** showed the highest inhibition against all the bacterial strains ranging from 41 to 165 μg/mL, as compared to the standards, streptomycin, kanamycin and vancomycin. Moreover, derivative **SKYa** was found to be the strongest against *M. tuberculosis* (83 μg/mL). Additionally, the anti-dengue potential of the coumarin hybrids as two-component NS2B/NS3 DENV flavivirus serine protease inhibitors was calculated using computational molecular docking approach, with reference to the standards 4-hydroxypanduratin, panduratin and ethyl 3-(4-(hydroxymethyl)-2-methoxy-5-nitrophenoxy)propanoate with DS of − 3.379, − 3.189 and − 3.381, respectively. The docking results revealed that the synthesized hybrids exhibited potent anti-dengue activity among which compounds **SKYf**, **SKYd**, **SKYc** and **SKYe** were found to be the best ones with docking scores of − 4.014, − 3.964, − 3.905 and − 3.889. In summary, we discovered 4-thiazolidinone coumarin derivatives as a new scaffold that may eventually yield useful compounds in the treatment of bacterial and viral infections.

**Electronic supplementary material:**

The online version of this article (10.1186/s13065-018-0435-0) contains supplementary material, which is available to authorized users.

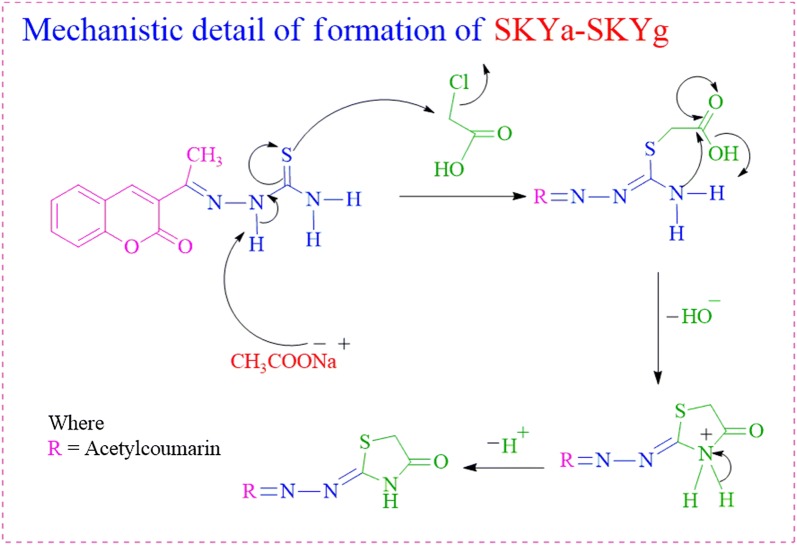

## Introduction

**Fig. 1:**
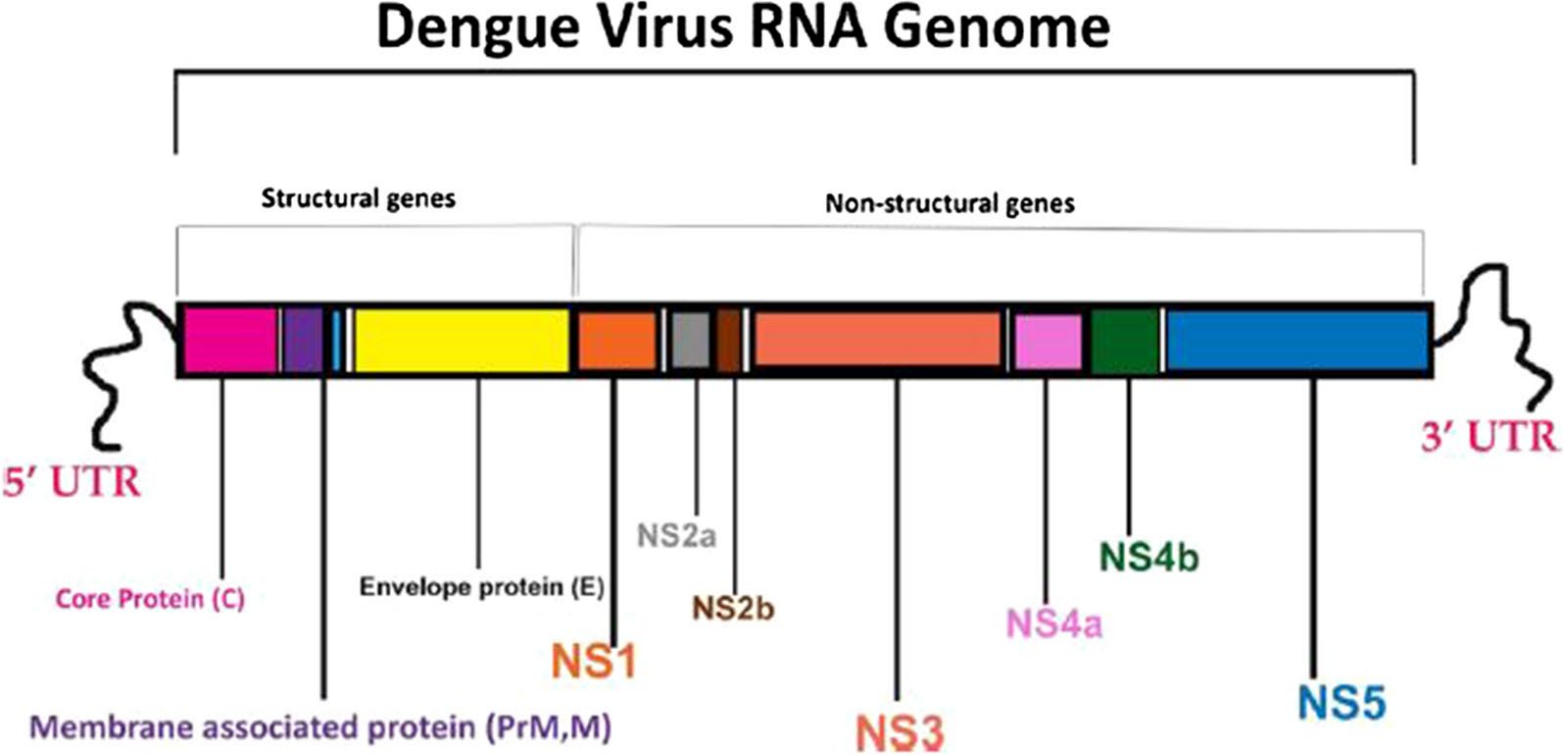
RNA genome of dengue virus. Structural and non-structural genes are indicated by various colors. Three structural proteins (Core protein, Envelope protein and Membrane associated protein) at 5′ UTR while seven non-structural proteins i.e. NS1, NS2a, NS2b, NS3, NS4a, NS4b and NS5 at 3′ UTR are represented [10, 11]

Bacteria are living organisms that possess only one cell. Through a microscope, they look like balls, rods, or spirals. Some bacteria helps in food digestion, can destroy disease-causing cells, and can provide the body with needed vitamins. However, infectious bacteria can affect us to serious level. They reproduce immensely fast in the body releasing off toxins, the chemical which can damage tissue and make us unwell. Examples of such bacteria are *Streptococcus*, *Staphylococcus*, *Acinetobacter* and *E. coli*, which give rise to the illness such as bacteraemia, pneumonia, meningitis, endocarditis, urinary tract infection and wound infections [1]. Antibiotics are the usual treatment for these. However, the problem of bacterial infection further gets complicated when coupled with the spread of antibiotic resistant bacteria [2, 3]. Though it is true that antibiotics and antimicrobials have revolutionized the treatment of infectious diseases, yet the rapid increase of antibiotics resistance has reached to a critical point. Bacteria have adapted defences against these antibiotics, even though we are developing newer drugs [4]. Another such serious infection is tuberculosis (TB), which is caused by highly pathogenic facultative intracellular bacterium called as *Mycobacterium tuberculosis* (MTB). According to World Health Organization (WHO), TB is among the second leading cause of death worldwide, as it is an easily spread air borne bacterial infection. Recent databases shoes approximately 9 million new cases and 1.5 million deaths owing to TB, including 360,000 deaths among HIV-positive people [5]. Furthermore, the emergence of multi drug resistance tuberculosis (MDR TB) and extensively drug resistant tuberculosis (XDR TB) has signalled the alarm in terms of the discovery of new potential anti-TB drugs. Concerns regarding to potential threats of such resistant strains to human health and wild life had become fatal over the passing years [6]. It is not too much to add here that following to this dengue is another serious re-emerging and resurging disease, which currently has no approved vaccines or antiviral therapies that can combat it. It is a mosquito-borne flavivirus infection, which could be caused by any of the four antigenically related serotypes viz, S-1, S-2, S-3 or S-4 [7]. In the year 2013 a fifth serotype S-5 has also been reported after screening the viral blood sample of a 37 years old Malaysian farmer, which on close analysis revealed that it was diverse from the rest four dengue serotypes and had some similarity with the dengue virus serotype-2 [8]. Taking an insight of the dengue virus (DENV) which is an ssRNA positive strand of virus, belongs to the family *Flaviviridae* together with some other important human pathogens such as Yellow Fever virus, West Nile virus and Japanese encephalitis virus and to the genus *Flavivirus* [9]. DENV has a genome, which comprises of nearly 11,000 bases, which codes three structural proteins viz: C, prM and E, seven nonstructural proteins viz: NS1, NS2A, NS2B, NS3, NS4A, NS4B and NS5 and short non-coding regions on its both ends viz: 5′ UTR and 3′ UTR (Fig. 1) [10, 11].

Coumarins are biologically active members of the benzopyrone family. Their derivatives are reported to display various biological activities such as as anti-bacterial [12, 13], anti-fungal [14–16], anti-coagulant [17], anti-dengue [18], anti-tuberculosis [19], anti-viral [20], anti-tumor [21, 22], anti-HIV [23] and anti-cytotoxicity [24]. Impressed by the strong biologically active profile of coumarin derivatives and as a part of our interest in the synthesis and screening of potentially bioactive compounds [19], we herein, report the synthesis of some novel 4-thiazolidinone coumarin hybrids (**SKYa**–**SKYg**) to be evaluated for their in vitro anti-bacterial, anti-tubercular activities, and as nonsubstrate based dengue virus NS2B/NS3 serine protease inhibitors via molecular docking approach. We targeted to study the structure–activity-relationship by altering the position of the substituents within the coumarin nucleus, as it is important to recognize the structural features in the coumarin nucleus for the design and development of new coumarin derivatives with remarkable biological activities.

## Results and discussion

### Chemistry

#### Synthesis of 4-thiazolidinone coumarin derivatives by application of Pearson’s HSAB principle

**Fig. 2:**
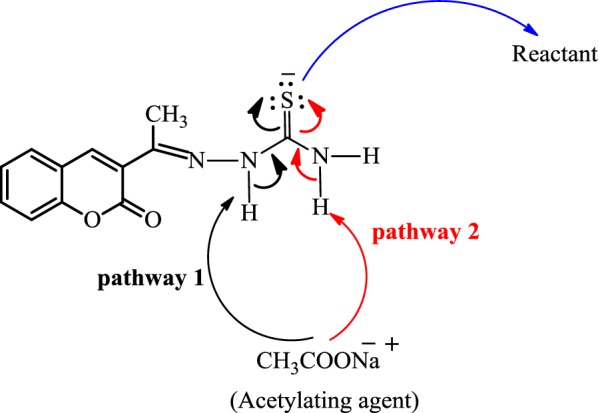
Acetylation possibilities of thiosemicarbazone moiety based on HSAB principle

There are several methods by which 4-thiazolidinone ring can be introduced to a coumarin skeleton such as acetylation of thiosemicarbazone, under different reaction conditions. Thiosemicarbazone has three nucleophilic centers, i.e. NH, NH_2_ and the sulphur atom. Cyclisation by acetylation using any acetylating agent could be achieved either by N atom of hydrazine with sulphur atom (pathway 1) or N atom of amino group with sulphur atom (pathway 2), depending on the Pearson’s HSAB principle, according to which hard acids prefer to coordinate hard bases and soft acids to soft bases (Fig. 2) [25].

**Scheme 1:**
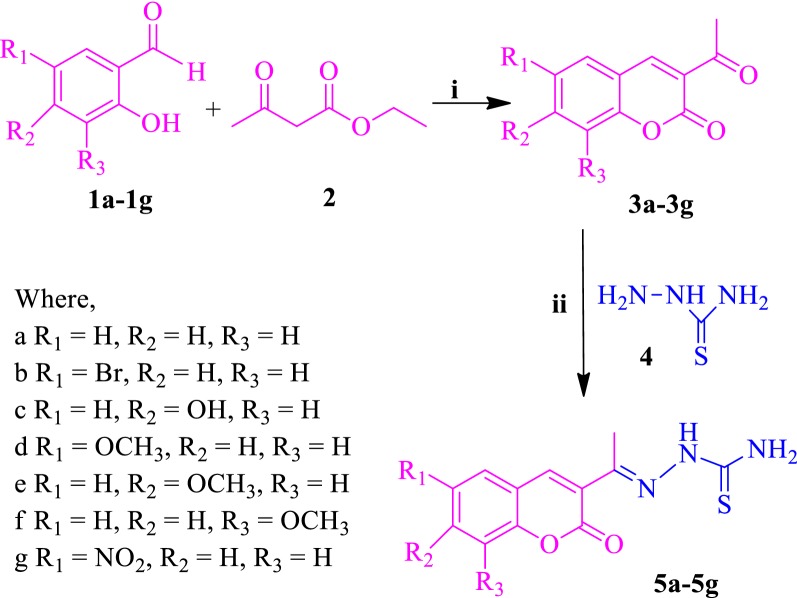
Synthesis of acetyl coumarin (**3a**–**3g**) and coumarin thiosemicarbazones (**5a**–**5g**). Reagent and reaction conditions: (i) piperidine, 0–5 °C (ii) CH_3_COOH, CH_3_OH, reflux

**Scheme 2:**
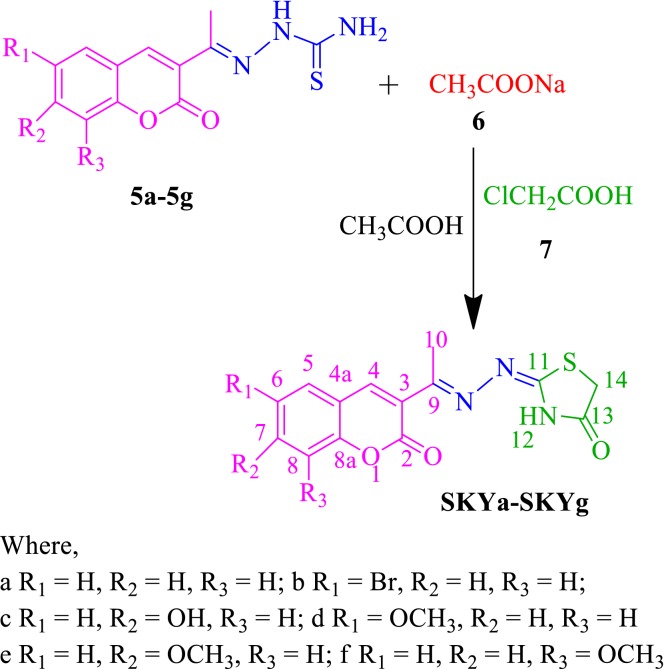
Synthesis of thiazolidinone coumarin derivatives (**SKYa**–**SKYg**). Reagent and solvent: CH_3_COONa/ClCH_2_COOH, CH_3_COOH, reflux

The synthesis of the designed compounds (**SKYa**–**SKYg**) was achieved in three steps. Condensation of salicylaldehyde (**1a**–**1g**) with ethylacetoacetate (**2**), at 0–5 °C in the presence of a catalytic amount of piperidine provided 3-acetylcoumarins (**3a**–**3g**) (Scheme 1). 3-Acetylcoumarins were further reacted with thiosemicarbazide (**4**) in methanol, to afford coumarin thiosemicarbazones (**5a**–**5g**) (Scheme 1). Corresponding coumarin thiosemicarbazone was reacted with sodium acetate (**6**), monochloroacetic acid (**7**) and few drops of acetic acid to furnish the desired compounds, **SKYa**–**SKYg** (Scheme 2).

**Fig. 3:**
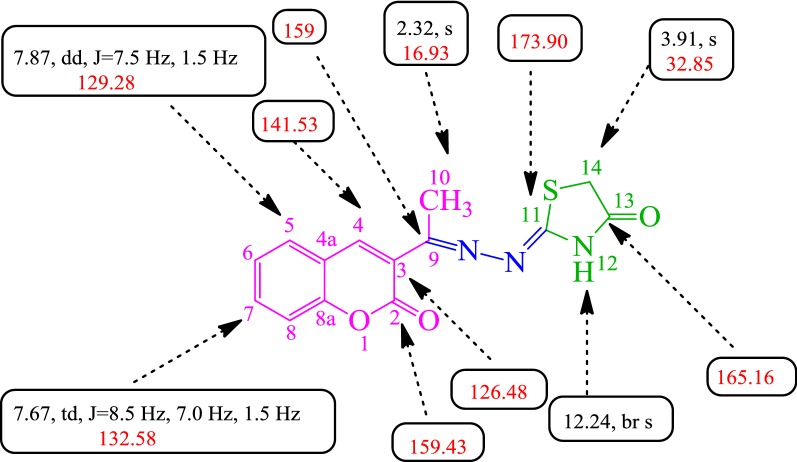
Selected ^1^H and ^13^C chemical shifts of **SKYa**

The structures of all the pure compounds (**SKYa**–**SKYg**) were elucidated by IR, ^1^H and ^13^C NMR spectroscopy, LC–MS and CHN analysis. The purity of all the compounds was checked by melting point measurements. The structure of the representative compound **SKYa** was further confirmed by 2D NMR spectroscopy (COSY, HMQC and HMBC), which gave exact configuration of the compound. The IR spectrum of the representative compound (Z)-2-((E)-(1-(2-oxo-2H-chromen-3-yl)ethylidene)hydrazono)thiazolidin-4-one **SKYa**, featured a sharp band at 3156.15/cm due to the NH stretching. The bands at 1723.06, 1626.05 and 1609.07/cm were corresponded to C=O lactone, C=O keto and C=N stretching, respectively. The elemental analysis (CHN) C, 55.86; H, 3.64; N, 13.90% confirmed the molecular formula as C_14_H_11_O_3_N_3_S. The LC–MS spectra indicated the molecular ion peak [MH]^+^, (+ESI) at *m/z* 302.0578 (301.0521) which further confirmed the molecular mass of the structure. The ^1^H NMR spectrum of the representative compound **SKYa**, revealed the presence of the two characteristic singlet of H-4 at *δ*_H_ 8.19 and NH at *δ*_H_ 12.24. On the other hand, a dd at *δ*_H_ 7.87 (J = 7.5, 1.5 Hz) was assigned to H-5 due to its ortho and meta coupling with H-6 and H-7, respectively. Whereas a td at *δ*_H_ 7.67 (J = 8.5, 7.0, 1.5 Hz) was assigned to H-7 due to its ortho coupling with H-6 and H-8 and meta coupling with H-5. In addition, a doublet at *δ*_H_ 7.45 (J = 8.0 Hz) was assigned to H-8 due to its ortho coupling with H-6 and a td at *δ*_H_ 7.40 (J = 8.5, 7.5, 0.5 Hz) was assigned to H-6 due to its ortho coupling with H-5, H-7 and meta coupling with H-8. Moreover, a broad singlet at *δ*_H_ 12.24 was assigned to NH. A sharp singlet at *δ*_H_ 3.91 was assigned to the methylene (CH_2_) protons of the thiazolidinone moiety, which was also further substantiated by ^1^H-^13^C HMQC, thus indicating the formation of thiazolidinone ring in the structure of **SKYa**. The ^13^C NMR spectrum of **SKYa**, showed expected signals corresponded to all 14 carbons in the structure. Three signals which were found to resonate at *δ*_C_ 173.90, 165.16 and 32.85 could be attributed to C-11, C-13 and C-14 of the thiazolidinone ring, which was further confirmed by 2D NMR. The lactone carbon, C-2 of coumarin, showed diagnostic chemical shift at *δ*_C_ 159.43 and the methyl carbon, C-10 was found to resonate at *δ*_C_ 16.93. Carbons of the coumarin core were found to resonate in the expected chemical shift regions with reference to those recorded for its analogue **3a**. The selected ^1^H and ^13^C chemical shifts are depicted in Fig. 3.

**Fig. 4:**
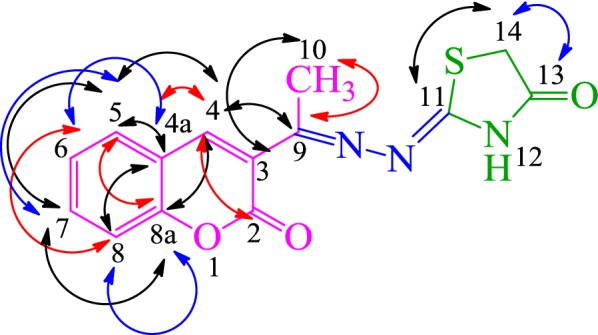
Possible ^1^H and ^13^C HMBC correlations of **SKYa**

Structure elucidation of **SKYa** was further substantiated by 2D-NMR spectroscopy. The ^1^H-^1^H COSY spectrum of **SKYa**, showed very clear correlations of H-5 (*δ*_H_ 7.87) with H-6 (*δ*_H_ 7.40) and a cross correlation of H-6 (*δ*_H_ 7.40) with H-7 (*δ*_H_ 7.67). H-7 (*δ*_H_ 7.67) was also found to correlate with H-8 (*δ*_H_ 7.45). The ^1^H-^13^C HMQC spectrum confirmed the connectivity of the protons relative to their respective carbons depicting that out of 14 carbons 7 were quaternary, 5 were methines, one was methylene and one was methyl carbon, as determined by DEPT 135 and DEPT 90 experiments. The spectrum clearly showed the coupling of H-4 (*δ*_H_ 8.19) with C-4 (*δ*_C_ 141.53), H-5 (*δ*_H_ 7.87) with C-5 (*δ*_C_ 129.28), H-6 (*δ*_H_ 7.40) with C-6 (*δ*_C_ 124.77), H-7 (*δ*_H_ 7.67) with C-7 (*δ*_C_ 132.58) and H-8 (*δ*_H_ 7.45) with C-8 (*δ*_C_ 115.99). The presence of thiazolidinone moiety was confirmed by a direct correlation of H-14 (*δ*_H_ 3.91) with C-14 (*δ*_C_ 32.85). The ^1^H-^13^C HMBC spectrum of **SKYa**, further substantiated the assignment of the aromatic carbons, in which long range correlations of C to H was observed. It was clearly shown that H-14 (*δ*_H_ 3.91) was correlated with C-13 (*δ*_C_ 165.16) and C-11 (*δ*_C_ 173.90) thus confirming the formation of a thiazolidinone ring. Furthermore, H-4 (*δ*_H_ 8.19) was found to correlate with C-9 (*δ*_C_ 159.0) and H-10 (*δ*_H_ 2.32) was found to correlate with C-9 (*δ*_C_ 159.0) and C-3 (*δ*_C_ 126.48) thus confirming the attachment of C-9 (*δ*_C_ 159.0) to C-3 (*δ*_C_ 126.48) of the coumarin nucleus. In addition, H-4 (*δ*_H_ 8.19) was also found to couple with C-8a (*δ*_C_ 153.47), C4a (*δ*_C_ 118.67), C-5 (*δ*_C_ 129.28) and C-2 (*δ*_C_ 159.43), which confirmed the presence of coumarin nucleus in the structure. H-5 (*δ*_H_ 7.87) was found to correlate with C4a (*δ*_C_ 118.67), C-7 (*δ*_C_ 132.58), C-4 (*δ*_C_ 141.53) and C-8a (*δ*_C_ 153.47). H-6 (*δ*_H_ 7.40) showed correlation with C-8 (*δ*_C_ 115.99) and C-4a (*δ*_C_ 118.67). H-7 (*δ*_H_ 7.67) showed correlation with C-5 (*δ*_C_ 129.28) and C-8a (*δ*_C_ 153.47). H-8 (*δ*_H_ 7.45) showed correlation with C-4a (*δ*_C_ 118.67), C-6 (*δ*_C_ 124.77) and C-8a (*δ*_C_ 153.47), respectively. The possible long-range interaction between the ^1^H and ^13^C atoms of **SKYa** are shown in Fig. 4 (see Additional file 1).

**Scheme 3:**
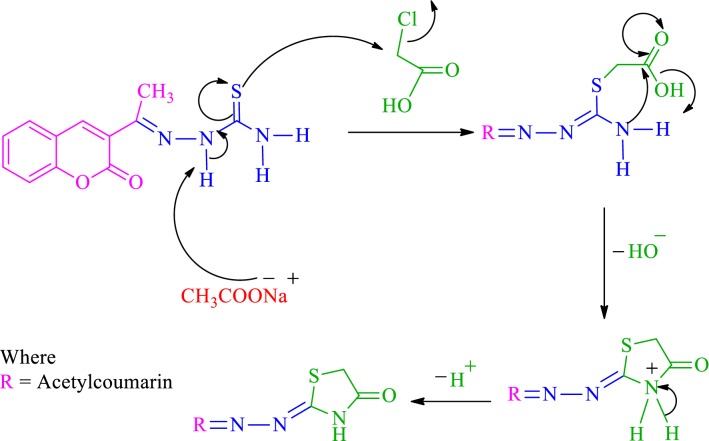
Plausible mechanism for the synthesis of 4-thiazolidinone coumarin (**SKYa**–**SKYg**)

The plausible mechanism for the formation of thiazolidinone could be summarised in two steps based on the HSAB principle. (i) The first step is S-alkylation of thiosemicarbazide in its thiol form, in the presence of sodium acetate. The removal of a proton from NH by sodium acetate resulted in the formation of an intermediate in a partially or totally thiol form, thus allowing the Soft–Soft interaction between S atom and electrophilic centre (CH_2_-Cl) [26, 27]. Therefore, this step involves nucleophilic attack by thiol on the electrophilic carbon of CH_2_-Cl to eliminate the leaving group Cl, which resulted in the S-alkylation of thiol and formation of a new C-S bond takes place. (ii) The second step is a Hard–Hard interaction between N atom of amino group (NH_2_) and carbonyl carbon, which resulted in an intramolecular cyclisation of the intermediate and the subsequent removal of a water molecule resulted in the formation of five membered thiazolidinone (Scheme 3) [25].

### Pharmacology

#### In-vitro anti-bacterial activity

**Fig. 5:**
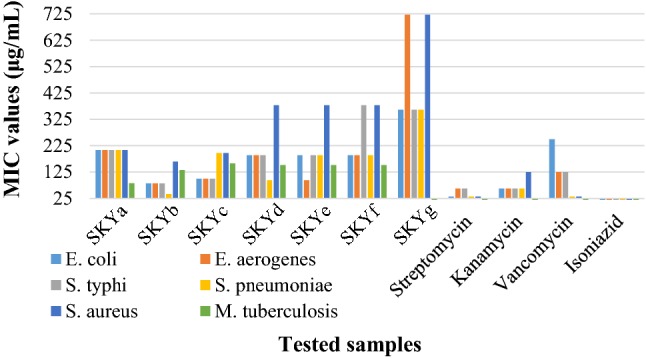
MIC (μg/mL) of the series of final compounds (**SKYa**–**SKYg**) against five different bacterial strains viz *E. coli*, *E. aerogenes*, *S. typhi*, *S. pneumoniae* and *S. aureus* and *M. tuberculosis* H37Rv strain ATCC 25618

The colorimetric microdilution assay was used to perform the in vitro anti-bacterial inhibitory activities and for the calculation of minimum inhibitory concentration (MIC) values (Fig. 5) of all the test compounds (**SKYa**–**SKYg**) against two Gram-positive bacteria (*Streptococcus pneumoniae* and *S. aureus*) and three Gram-negative bacteria (*E. coli, Enterobacter aerogenes* and *Salmonella typhi*) with reference to the standard drugs streptomycin, kanamycin, and vancomycin. Interestingly, it was found that all the tested coumarin derivatives exhibited quite good to moderate inhibition ranging between 31.25 and 250 μg/mL. Potent inhibitory activity against all the pathogens was observed by compound **SKYb** with MIC values of 41–165 μg/mL followed by compound **SKYc** and **SKYd**. The inhibitory activities of the compound **SKYb** especially against *E. aerogenes* and *S. pneumoniae*, was comparable and even better than that of standard drugs vancomycin and kanamycin, respectively. Compound **SKYe** showed very good inhibition of 94 μg/mL against *E. aerogenes* as compared to rest of the compounds, which was even higher than the standard vancomycin. It also exhibited good potency for the pathogen, *E. coli* with MIC value of 189 μg/mL. Compounds **SKYd**, **SKYe** and **SKYf** of the series exhibited lower MIC even from the reference drug vancomycin against *E. coli*. It is worth mentioning here, that the introduction of halogen, hydroxyl group and methoxy group, in coumarin skeleton for the compounds **SKYb**, **SKYc** and **SKYd** enhanced the power of bacterial inhibition against most of the tested strains when compared to other substituents (Table 1). Further improvement on the substitution pattern is being carried out to increase the potential of these derivatives as anti-bacterial agents (Additional file 1).

**Table 1:** Anti-bacterial and anti-tuberculosis activities (MIC, μg/mL) of coumarin hybrids (**SKYa**–**SKYg**)

Compounds	*E. coli*	*E. aerogenes*	*S. typhi*	*S. pneumoniae*	*S. aureus*	*M. tuberculosis*
**SKYa**	208	208	208	208	208	83
**SKYb**	82	82	82	41	165	132
**SKYc**	99	99	99	197	197	158
**SKYd**	189	189	189	94	378	151
**SKYe**	189	94	189	189	378	151
**SKYf**	189	189	378	189	378	151
**SKYg**	361	722	361	361	722	–
Streptomycin	*31.25*	*62.5*	*62.5*	*31.25*	*31.25*	–
Kanamycin	*62.5*	*62.5*	*62.5*	*62.5*	*125*	–
Vancomycin	*250*	*125*	*125*	*31.25*	*31.25*	–
Isoniazid	–	–	–	–	–	*0.0781*

Results are mean of triplicate values (n = 3)

Italic values indicate standard use

– Not applicable

No inhibition observed after highest test concentration of 50 μg/mL for *M. tuberculosis*

#### In-vitro anti-tuberculosis activity

To complete the multi-target biological profile of the test compounds (**SKYa**–**SKYg**), the in vitro anti-TB inhibitory activity against *M. tuberculosis*, H37Rv strain ATCC 25618 was measured with reference to the control drug isoniazid. All the test compounds except **SKYg** exhibited anti-TB activity with the highest chosen concentration level of 50 μg/mL. Results obtained for **SKYb**, **SKYd**, **SKYe** and **SKYf** indicated that the introduction of halogen and methoxy group could enhance the anti-TB activity. It was also apparent from the results that the introduction of hydroxyl could also exert considerable anti-TB activity as shown by compounds **SKYc** with MIC values of 132, 151 and 158 μg/mL, respectively. Compound **SKYg** showed no significant inhibitory activity, indicating that all compounds are clearly selective inhibitors and that the presence of nitro group had no inhibitory effects on the tubercle cells even at the highest concentration range of 50 μg/mL. Compared to the standard, these active compounds fared moderately (Fig. 5). Concerning anti-TB activity, compounds possessing MIC values of 1.5 μg/mL are considered promising [28]. However, these compounds might not be drugs per se if they are toxic, insoluble or pharma kinetically limited. Noticeably, the structural differences of the compounds could provide ideas for the designing of new anti-microbial agents. Therefore, the structural skeleton of compounds **SKYb**, **SKYc** and **SKYd** could also provide a useful template for the development of new anti-TB drugs (Table 1).

#### Structure–activity-relationship (SAR) analysis

Substituents play a very important role in the bio-activity of any molecule. The position and site of attachment of the group (for example; thiazole ring, halogen, methyl, methoxy, hydroxyl, nitro and amino substituents) and its electronic nature contributes profoundly to its bioactive profile [29]. The SAR reveals that physiochemical properties such as lipophilicity or hydrophobicity and electronegativity of any substituent effectively controls its bio-activeness towards any pathogen. The more hydrophobic the substituent, the more effective are its antibacterial and anti-tubercular properties. Bio-activity of 4-thiazolidinone-coumarins seems to increase 4 to 8 folds more in the presence of halogen or hydroxyl groups (good hydrophobic), when compared to the standards streptomycin, kanamycin and vancomycin. In the presence of OCH_3_ group (moderate hydrophobic), the bio-activeness was reported as between good and moderate and NO_2_ group (hydrophilic), was found to exhibit relatively lower bio-activity. A quite satisfactory explanation behind this, is the high electronegativity and high effective nuclear charge of the halogens which make them quite reactive and thus they tend to increase the lipophilicity or hydrophobicity of the molecules, making them bigger, more polarized and accordingly increasing the London dispersion forces, which are responsible for the interaction of the lipophilic substance to themselves or with others. Alcoholic hydroxyl groups (-OH), are quite polar and hence hydrophilic (water loving) in nature. But, is should be noted that their carbon chain portion is non-polar which makes them hydrophobic, overall more nonpolar and therefore less soluble in the polar water as the carbon chain grows. The methoxy group (OCH_3_) on the other hand has little influence on the molecular hydrophobicity and its bio-activities are between good and moderate. Opposite to these the nitro functional groups (NO_2_) are hydrophilic which form strong hydrogen bonds with water molecules, despite of their high polarities arising due to large dipole moments. As a result, these compounds are hydroneutral, with hydrophilicity between hydrophilic and hydrophobic [30]. The overall results showed, that the bio-activities of the tested compounds increased several times with the halogen or hydroxyl groups in the coumarin skeleton (**SKYb**, **SKYc**). Whereas the activity was in between good to moderate in the presence of OCH_3_ (**SKYd**, **SKYe**, **SKYf**) and NO_2_ group (**SKYg**). Therefore, it could be concluded that by replacing or changing the groups in the coumarin pharmacophore could result in better structural modifications of the molecule making them display even more better bio-activities.

#### Molecular docking

The molecular docking methodology can provide a greater understanding of the ligand–protein interactions. With this motive, all the synthesized compounds were docked into the active site of enzyme. Docking against the dengue virus NS2B/NS3 protease helps immensely in the prediction of their interaction ability. For results comparison, 4-hydroxypanduratin (DS − 3.379), panduratin (DS − 3.189) and ethyl 3-(4-(hydroxymethyl)-2-methoxy-5-nitrophenoxy)propanoate (DS − 3.381) were docked as positive controls. The 3D crystallographic structure of DENV NS2B/NS3 protease was obtained from PDB (PBD ID: 2FOM), at a resolution of 1.50 Å. The aim is to target the hydrophobic pockets of dengue virus NS2B/NS3 protease, and to screen all compounds that could help in the inhibition of DENV infection. The results thus could offer useful information in the development of drug and would further help in computer-aided drug designing, against the DENV infection. Dengue virus possess of four antigenically related serotypes, such as dengue S-1, S-2, S-3 and S-4 [7, 31] and interestingly any of the inhibitor could act against these serotypes, in the binding pocket of NS2B/NS3 protease [32]. Heavy number of envelope proteins surrounds the mature dengue virus at its surface, hence initiating the points for the systematic search of cavities to help discover those compounds that could interfere in the E protein rearrangements, which results in fusion process [33]. Like other flavivirus, dengue virus has also been specified as a significant drug target. As its catalytic triad is already known to be quite important in viral replication, therefore any disruption in it could block the replication of the DENV [34].

**Fig. 6:**
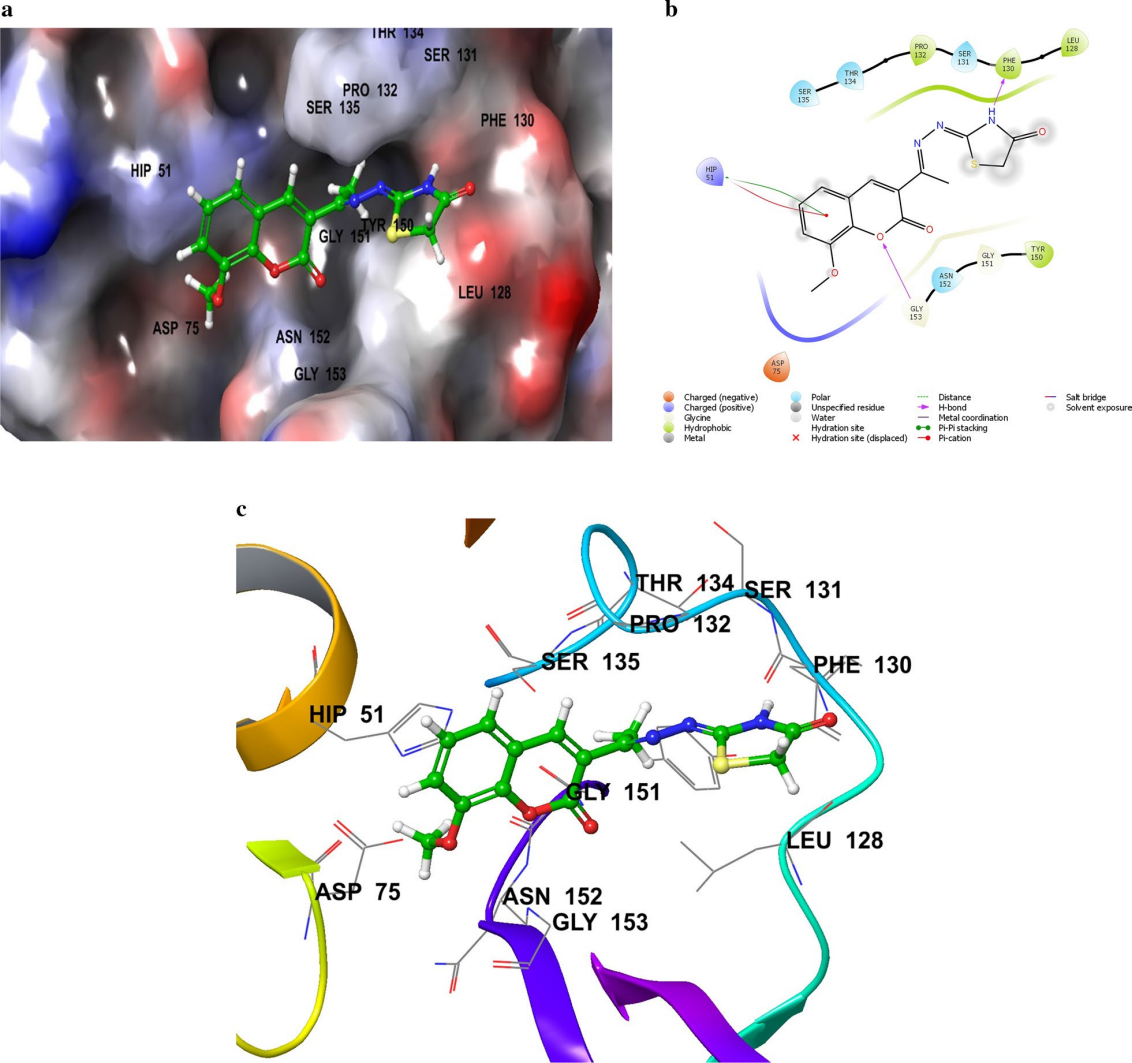
Binding modes of compound **SKYf** with dengue virus NS2B/NS3 protease: **a** Protein as surface and compound as sticks; **b** Protein and compound as 2D interaction image; **c** Protein as ribbon and compound as sticks

**Fig. 7:**
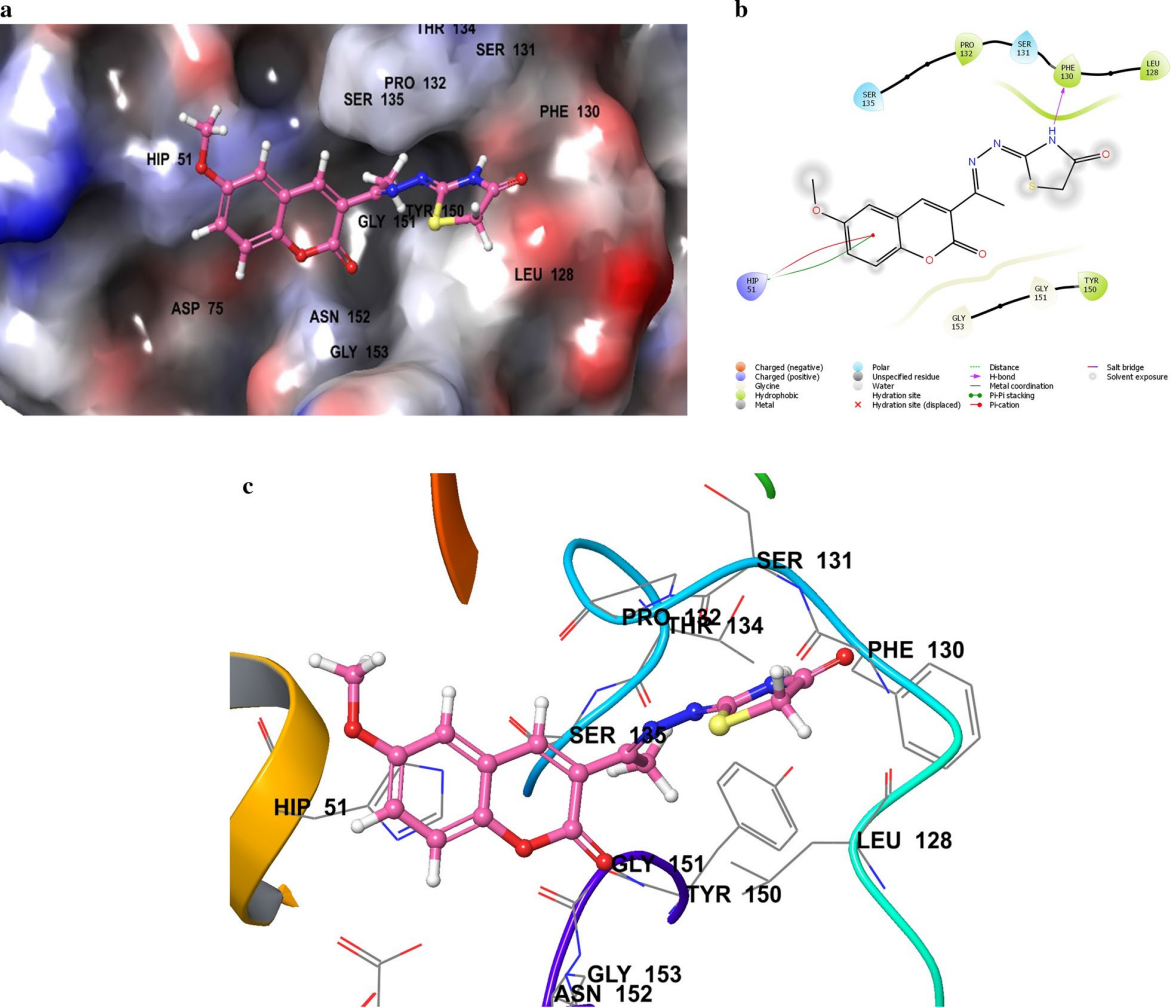
Binding modes of compound **SKYd** with dengue virus NS2B/NS3 protease: **a** Protein as surface and compound as sticks; **b** Protein and compound as 2D interaction image; **c** Protein as ribbon and compound as sticks

**Fig. 8:**
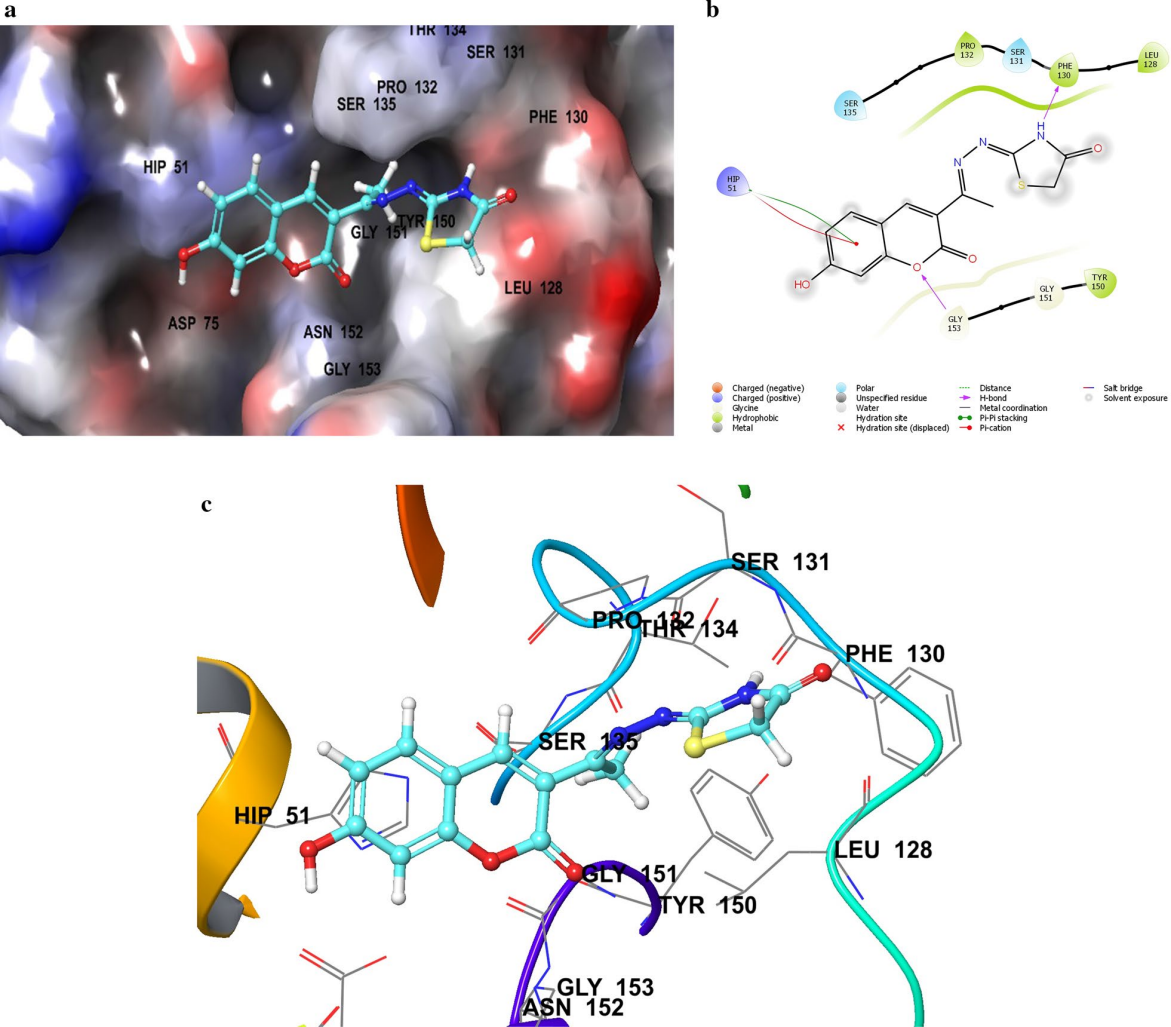
Binding modes of compound **SKYc** with dengue virus NS2B/NS3 protease: **a** Protein as surface and compound as sticks; **b** Protein and compound as 2D interaction image; **c** Protein as ribbon and compound as sticks

**Fig. 9:**
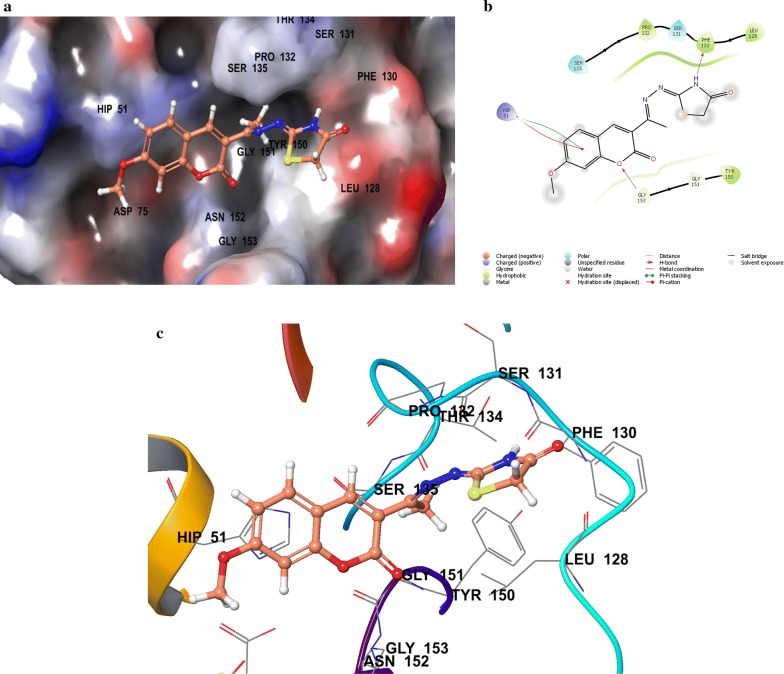
Binding modes of compound **SKYe** with dengue virus NS2B/NS3 protease: **a** Protein as surface and compound as sticks; **b** Protein and compound as 2D interaction image; **c** Protein as ribbon and compound as sticks

Compounds **SKYa**–**SKYg**, were interacted with the residues in the catalytic triad, such as HIS51, ASP75 and SER135 of the protease. Lee and co-workers reported that these residues forms hydrogen bond with the active ligands, through the carbonyl group of GLY151 and the hydroxyl group of SER135, but no interaction was reported with the HIS51 of the catalytic triad [35]. All docked compounds (**SKYa**–**SKYg**), were observed to occupy similar poses with binding orientation around the active sites of the protease NS2B/NS3, with different interactions with the residues within a range of (DS − 2.754 to − 4.014) (Table 2). The most active compound **SKYf**, showed quite high binding affinity (high negative docking score –4.014), even higher than that of the chosen standards, with hydrogen bond, π-π stacking and π-cation interactions. Interestingly, the binding affinity of the most active compounds (**SKYf**, **SKYd**, **SKYc** and **SKYe**) increase with the present of these interactions with the most important residues inside the active site, such as HIS51, ASP75, GLY151, and GLY153 (see Figs. 6, 7, 8 and 9).

**Table 2:** Compounds (**SKYa**–**SKYg**), docking score, interacting residues and close contact residues

Comp	Docking score	Interacting residues	Close contact residues
**SKYa**	− 2.754	–	HIS51, ASP75, TYR150, GLY151, ASN152, GLY153, SER135, PRO132, SER131, PHE130, LEU128
**SKYb**	− 2.960	HIS51 (H bond) and (two π-cation)	ASP75, VAL154, GLY153, ASN152, GLY151, TYR150, HIS51, LEU128, PHE130, SER131, PRO132, SER135
**SKYc**	− 3.905	GLY153 (H bond), PHE130 (H bond), HIS51 (π–π stacking and π-cation)	HIS51, GLY153, GLY151, TYR150, LEU128, PHE130, SER131, PRO132, SER135
**SKYd**	− 3.964	PHE130 (H bond), HIS51 (π–π stacking and π-cation)	HIS51, GLY153, GLY151, TYR150, LEU128, PHE130, SER131, PRO132, SER135
**SKYe**	− 3.889	GLY153 (H Bond), PHE130 (H Bond), HIS51 (π–π stacking and π-cation)	GLY153, GLY151, TYR150, LEU128, PHE130, SER131, PRO132, SER135
**SKYf**	− 4.014	GLY153 (H bond), PHE130 (H bond), HIS51 (π–π stacking and π-cation)	HIS51, ASP75, GLY153, ASN152, GLY151, TYR150, LEU, PHE130, SER131, PRO132, THR134, SER135
**SKYg**	− 2.992	GLY151 and LEU128 (H bond)	HIS51, ASP75, GLY153, ASN152, GLY151, TYR150, SER135, PRO132, SER131, PHE130, ASP129, LEU128

**Fig. 10:**
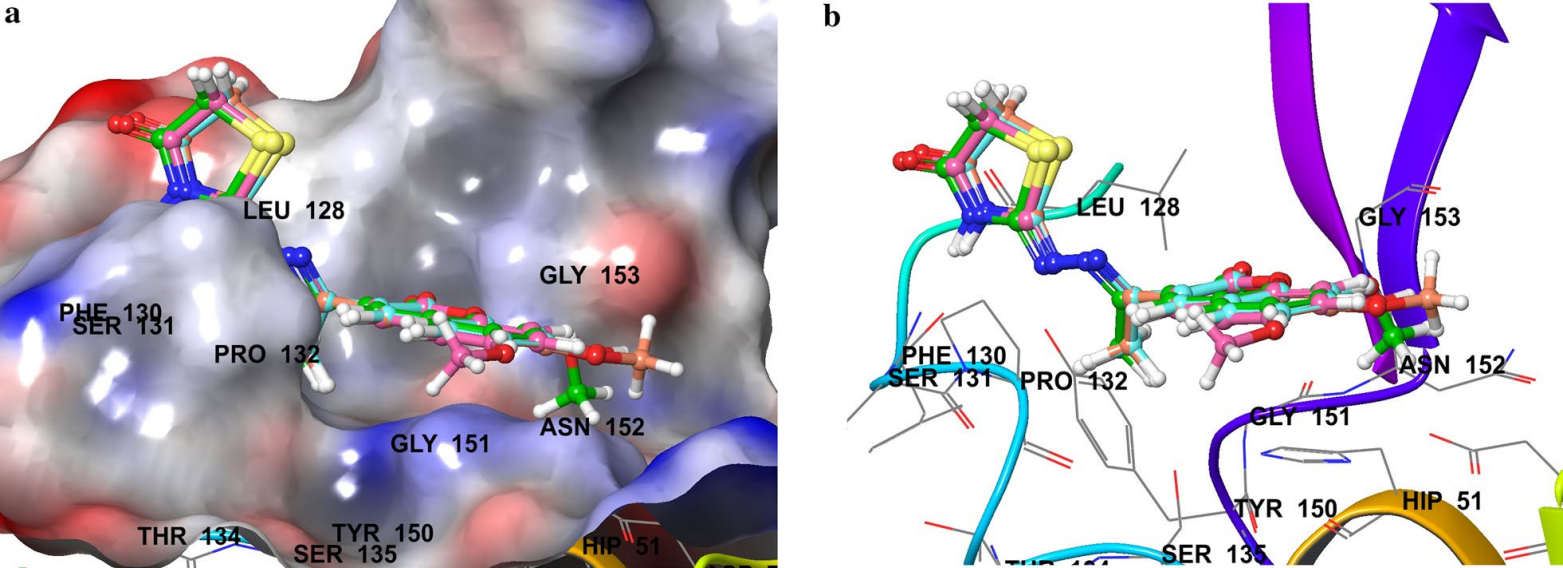
Binding modes of compounds **SKYf**, **SKYd**, **SKYc** and **SKYe** with dengue virus NS2B/NS3 protease: **a** Protein as surface and compounds as sticks; **b** Protein as ribbon and compound as sticks

Inside active site, the orientation of the four most active compounds (**SKYf**, **SKYd**, **SKYc** and **SKYe**), was directed by keeping coumarin group towards inside and thiazolidin-4-one group towards outside (Fig. 10). It is important to mention that the substitution of methoxy and hydroxy groups at 6, 7 or 8 positions of the coumarin scaffold, increases the binding ability more as compared to the other groups. The possible effects of these groups as strong electron donating groups was clear at position 8 displaying highest DS (compound **SKYf**), and lowest effect with least DS at position 7 (compound **SKYe**). Moreover, other substitutions (Br and NO_2_) caused different orientation of each compound’s directions, but interacted with most of the important residue inside the active site. The possible effects of these groups were either as strong electron withdrawing groups or to increase coumarin size in order to make it interact to the close and nearby important residues. Further recommended substitution, could be at position 8, with any electron-donating group to enhance the binding ability. The identification of thiazolidinone coumarin hybrids as potent lead compounds as the desired hotspot inhibitors clearly reflects the significance of this study.

## Materials and methods

### Chemistry

Solvents and reagents of analytical grade were purchased from Sigma-Aldrich, ACROS Organics and Merck and used as it is unless otherwise stated, the normal workup from organic solvent involved drying over Na_2_SO_4_, MgSO_4_ and rotary evaporation. TLC was performed on aluminium-backed Merck Silica Gel 60 F-254 sheets using suitable solvent systems with spots being visualized by a UV Lamp (254 or 365 nm). Deuterated solvents were used as received. Melting points were obtained in open capillary tubes using a Stuart Scientific (SMP-1) instrument and were uncorrected. The FTIR spectra was recorded using Perkin Elmer FTIR-ATR spectrometer Frontier as KBr pellets at the wavelength of 4000–650/cm. 1D and 2D NMR spectra were recorded on a Bruker Avance 500 FT-NMR instrument at 500 MHz for ^1^H and 2D NMR experiments (COSY, HMQC and HMBC) and at 125 MHz for ^13^C NMR, DEPT 90 and DEPT 135 experiments, using TMS as internal standard and DMSO-d_6_ as solvent. Bruker Topspin software v 3.0 was used to process the NMR raw data. Chemical shifts were expressed in parts per million on δ scale and the coupling constants were given in Hertz (Hz). Mass spectra were conducted on an Agilent Technologies 6224 TOF LC–MS spectrometer. The measurements were carried out in positive mode. Elemental analyses were accomplished on a Perkin Elmer 2400 series Elemental CHN analyzer and were within ± 0.3% of the theoretical values. Automated docking studies for dengue were carried out using the Maestro™ software package (v. 12.1, Schrödinger, LLC, New York, NY, 2011) program.

#### General procedure for the synthesis of 3-acetylcoumarins (**3a**–**1g**) and coumarin thiosemicarbazones (**5a**–**5g**)

To a cooled mixture of salicylaldehyde derivatives (**1a**–**1g**) (0.20 mol) and ethyl acetoacetate **2** (0.25 mol) a catalytic amount of piperidine was added with continuous stirring. The reaction mixture was rested for 12 h, resulting in the formation of a yellow solid which was washed with cold ether and recrystallized by ethanol/CHCl_3_ (1:3, v/v) mixtures, to afford pure 3-acetylcoumarins (**3a**–**3g**) as fine yellow needles in good yields. Thiosemicarbazide (**4**) (2.8 mmol) was added to the methanolic solution of the series of corresponding acetyl coumarin (**3a**–**3g**) (2.8 mmol), along with a few drops of glacial acetic acid. After 4 h of refluxing, the precipitate was filtered and washed with cold water. Recrystallization from ethanol/ethyl acetate (2:1, v/v) afforded good yields of coumarin thiosemicarbazones (**5a**–**5g**) (Scheme 1) [19].

#### General procedure for the synthesis of 4-thiazolidinone coumarin hybrids (**SKYa**–**SKYg**)

A mixture of various corresponding coumarin thiosemicarbazone (**5a**–**5g**) (0.01 mol), anhydrous sodium acetate (**6**) (0.01 mol) and monochloroacetic acid (**7**) (0.01 mol) in absolute ethanol (20 mL) was heated under reflux for 5 h with continuous stirring. Initially a clear solution was formed which on slow evaporation of excess solvent gave whitish solid. Work-up and recrystallization from EtOH/water afforded the target compounds (**SKYa**–**SKYg**) as white colour solids in good yields **(**Scheme 2) [25].

##### *(Z)*-*2*-*((E)*-*(1*-*(2*-*Oxo*-*2H*-*chromen*-*3*-*yl)ethylidene)hydrazono)thiazolidin*-*4*-*one (****SKYa****)*

White solid, (1.90 g, 63.1%), mp 256–258 °C. IR KBr (ν_max_/cm^−1^): 3156.15 (N–H), 1723.06 (C=O lactone), 1626.05 (C=O keto), 1609.07 (C=N); ^1^H NMR (δ/ppm, 500 MHz, DMSO-*d*_*6*_): 12.24 (1H, br s, N–H), 8.19 (1H, s, H-4), 7.87 (1H, dd, *J* = 7.5, 1.5 Hz, H-5), 7.67 (1H, td, *J* = 8.5, 7.0, 1.5 Hz, H-7), 7.45 (1H, d, *J* = 8.0 Hz, H-8), 7.40 (1H, td, *J* = 7.5, 0.5 Hz, H-6), 3.91 (2H, s, H-14), 2.32 (3H, s, CH_3_); ^13^C NMR (δ/ppm, 125 MHz, DMSO-*d*_*6*_): 173.90 (C-11), 165.16 (C-13), 159.43 (C-2), 159.00 (C-9), 153.47 (C-8a), 141.53 (C-4), 132.58 (C-7), 129.28 (C-5), 126.48 (C-3), 124.77 (C-6), 118.67 (C-4a), 115.99 (C-8), 32.85 (C-14), 16.93 (CH_3_). Anal. Calcd. For C_14_H_11_O_3_N_3_S (301.32/gmol): C, 55.80; H, 3.68; N, 13.95%. Found: C, 55.86; H, 3.64; N, 13.90%. MS (+ESI) (*m/z*): 302.0578 (301.0521).

##### *(Z)*-*2*-*((E)*-*(1*-*(6*-*Bromo*-*2H*-*chromen*-*3*-*yl)ethylidene)hydrazono)thiazolidin*-*4*-*one (****SKYb****)*

White solid, (2.57 g, 67.6%), mp 249–251 °C. IR KBr (ν_max_/cm^−1^): 3152.26 (N–H), 1733.03 (C=O lactone), 1690.11 (C=O keto), 1622.09 (C=N); ^1^H NMR (δ/ppm, 500 MHz, DMSO-*d*_*6*_): 12.23 (1H, br s, N–H), 8.16 (2H, s, H-4 & H-5), 7.80 (1H, dd, *J* = 9.0, 205 Hz, H-7), 7.42 (1H, d, *J* = 8.5 Hz, H-8), 3.90 (2H, s, H-14), 2.30 (3H, s, CH_3_); ^13^C NMR (δ/ppm, 125 MHz, DMSO-*d*_*6*_): 174.17 (C-11), 165.88 (C-13), 158.93 (C-2), 158.54 (C-8a), 152.52 (C-9), 140.14 (C-4), 134.75 (C-7), 131.22 (C-5), 127.55 (C-3), 120.63 (C-6), 118.25 (C-8), 116.25 (C-4a), 32.95 (C-14), 16.87 (CH_3_). Anal. Calcd. For C_14_H_10_O_3_N_3_SBr (380.22/gmol): C, 44.22; H, 2.65; N, 11.05%. Found: C, 44.18; H, 2.69; N, 11.00%. MS (+ESI) (*m/z*): 381.0902 (378.9626).

##### *(Z)*-*2*-*((E)*-*(1*-*(7*-*Hydroxy*-*2H*-*chromen*-*3*-*yl)ethylidene)hydrazono)thiazolidin*-*4*-*one (****SKYc****)*

White solid, (2.48 g, 78.2%), mp 261–263 °C. IR KBr (ν_max_/cm^−1^): 3450.20 (O–H), 3096.74 (N–H), 1723.63 (C=O lactone), 1693.97 (C = O keto), 1625.15 (C=N); ^1^H NMR (δ/ppm, 500 MHz, DMSO-*d*_*6*_): 11.71 (1H, br s, N–H), 8.10 (1H, s, H-4), 7.69 (1H, d, *J* = 9.5 Hz, H-5), 6.84 (1H, dd, *J* = 8.5, 2.5 Hz, H-6), 6.76 (1H, d, *J* = 2.5 Hz, H-8), 3.88 (2H, s, H-14), 3.35 (1H, s, O–H), 2.30 (3H, s, CH_3_); ^13^C NMR (δ/ppm, 125 MHz, DMSO-*d*_*6*_): 173.91 (C-11), 162.05 (C-13), 159.40 (C-2), 157.67 (C-8a), 155.65 (C-9), 142.11 (C-4), 130.80 (C-7), 129.26 (C-5), 121.64 (C-4a), 111.17 (C-3), 101.79 (C-6), 66.32 (C-8), 32.80 (C-14), 23.12 (CH_3_). Anal. Calcd. For C_14_H_11_O_4_N_3_S (317.32/gmol): C, 52.99; H, 3.49; N, 13.24%. Found: C, 53.03; H, 3.53; N, 13.28%. MS (+ESI) (*m/z*): 318.2988.

##### *(Z)*-*2*-*((E)*-*(1*-*(6*-*Methoxy*-*2H*-*chromen*-*3*-*yl)ethylidene)hydrazono)thizolidin*-*4*-*one (****SKYd****)*

White solid, (2.54 g, 76.7%), mp 248–250 °C. IR KBr (ν_max_/cm^−1^): 3143.76 (N–H), 1721.04 (C=O lactone), 1702.14 (C=O keto), 1621.37 (C=N); ^1^H NMR (δ/ppm, 500 MHz, DMSO-*d*_*6*_): 12.10 (1H, br s, N–H), 8.13 (2H, s, H-4), 7.44 (1H, d, *J* = 3.0 Hz, H-5), 7.39 (1H, d, *J* = 9.0 Hz, H-7), 7.42 (1H, d, *J* = 9.0, 3.0 Hz, H-8), 3.88 (2H, s, H-14), 3.82 (3H, s, OCH_3_), 2.30 (3H, s, CH_3_); ^13^C NMR (δ/ppm, 125 MHz, DMSO-*d*_*6*_): 173.93 (C-11), 164.75 (C-13), 163.08 (C-2), 159.50 (C-8a), 159.26 (C-9), 155.54 (C-4), 141.81 (C-7), 130.45 (C-5), 122.68 (C-3), 112.89 (C-6), 112.24 (C-4a), 100.32 (C-8), 56.03 (OCH_3_), 32.83 (C-14), 16.92 (CH_3_). Anal. Calcd. For C_15_H_13_O_4_N_3_S (331.35/gmol): C, 54.37; H, 3.95; N, 12.68%. Found: C, 54.41; H, 3.91; N, 12.64%. MS (+ESI) (*m/z*): 332.0697 (331.0626).

##### *(Z)*-*2*-*((E)*-*(1*-*(7*-*Methoxy*-*2H*-*chromen*-*3*-*yl)ethylidene)hydrazono)thiazolidin*-*4*-*one (****SKYe****)*

White solid, (2.62 g, 79.1%), mp 259–261 °C. IR KBr (ν_max_/cm^−1^): 3120.09 (N–H), 1726.12 (C=O lactone), 1715.02 (C=O keto), 1612.77 (C=N); ^1^H NMR (δ/ppm, 500 MHz, DMSO-*d*_*6*_): 12.00 (1H, br s, N–H), 8.12 (2H, s, H-4), 7.79 (1H, d, *J* = 9.0 Hz, H-5), 7.04 (1H, d, *J* = 2.0 Hz, H-8), 6.99 (1H, dd, *J* = 9.0, 2.5 Hz, H-6), 3.88 (3H, s, OCH_3_), 3.87 (2H, s, H-14), 2.31 (3H, s, CH_3_); ^13^C NMR (δ/ppm, 125 MHz, DMSO-*d*_*6*_): 173.93 (C-11), 164.75 (C-13), 163.08 (C-2), 159.50 (C-8a), 159.26 (C-9), 155.54 (C-4), 141.81 (C-7), 130.45 (C-5), 122.68 (C-3), 112.89 (C-6), 112.24 (C-4a), 100.32 (C-8), 56.03 (OCH_3_), 32.83 (C-14), 16.92 (CH_3_). Anal. Calcd. For C_15_H_13_O_4_N_3_S (331.35/gmol): C, 54.37; H, 3.95; N, 12.68%. Found: C, 54.33; H, 4.0; N, 12.64%. MS (+ESI) (*m/z*): 332.0705 (331.0626).

##### *(Z)*-*2*-*((E)*-*(1*-*(8*-*Methoxy*-*2H*-*chromen*-*3*-*yl)ethylidene)hydrazono)thiazolidin*-*4*-*one****(SKYf****)*

White solid, (2.70 g, 81.6%), mp 269–271 °C. IR KBr (ν_max_/cm^−1^): 3233.60 (N–H), 1731.48 (C=O lactone), 1684.00 (C=O keto), 1608.48 (C=N); ^1^H NMR (δ/ppm, 500 MHz, DMSO-*d*_*6*_): 12.01 (1H, br s, N–H), 8.16 (2H, s, H-4), 7.41 (1H, dd, *J* = 7.5, 2.0 Hz, H-5), 7.31–7.37 (2H, m, H-6 & H-7), 3.94 (3H, s, OCH_3_), 3.89 (2H, s, H-14), 2.32 (3H, s, CH_3_); ^13^C NMR (δ/ppm, 125 MHz, DMSO-*d*_*6*_): 173.88 (C-11), 165.19 (C-13), 159.37 (C-2), 158.71 (C-8a), 146.29 (C-9), 142.84 (C-4), 141.72 (C-7), 26.62 (C-4a), 124.70 (C-5), 120.39 (C-6), 119.25 (C-3), 114.80 (C-8), 56.15 (OCH_3_), 32.85 (C-14), 16.92 (CH_3_). Anal. Calcd. For C_15_H_13_O_4_N_3_S (331.35): C, 54.37; H, 3.95; N, 12.68%. Found: C, 54.33; H, 4.0; N, 12.64%. MS (+ESI) (*m/z*): 332.0696 (331.0626).

##### *(Z)*-*2*-*((E)*-*(1*-*(6*-*Nitro*-*2H*-*chromen*-*3*-*yl)ethylidene)hydrazono)thiazolidin*-*4*-*one(****SKYg****)*

White solid, (2.73 g, 78.9%), mp 235–237 °C. IR KBr (ν_max_/cm^−1^): 3134.72 (N–H), 1732.12 (C=O lactone), 1683.40 (C=O keto), 1622.59 (C=N); ^1^H NMR (δ/ppm, 500 MHz, DMSO-*d*_*6*_): 12.21 (1H, br s, N–H), 8.11 (1H, s, H-4), 7.39 (1H, d, *J* = 2.5 Hz, H-5), 7.79 (1H, dd, *J *= 8.5, 2.0 Hz, H-7), 7.41 (1H, d, *J* = 8.0 Hz, H-8), 3.92 (2H, s, H-14), 2.32 (3H, s, CH_3_); ^13^C NMR (δ/ppm, 125 MHz, DMSO-*d*_*6*_): 173.12 (C-11), 163.00 (C-13), 157.23 (C-2), 158.45 (C-8a), 151.52 (C-9), 140.14 (C-4), 134.67 (C-7), 131.20 (C-5), 126.55 (C-3), 120.23 (C-6), 119.27 (C-8), 117.78 (C-4a), 35.89 (C-14), 19.85 (CH_3_). Anal. Calcd. For C_14_H_10_O_5_N_4_S (346.32): C, 48.55; H, 2.91; N, 16.18%. Found: C, 48.85; H, 2.87; N, 16.22%. MS (+ESI) (*m/z*): 347.0403 (346.0371).

### Pharmacological evaluation

#### In-vitro evaluation of anti-bacterial activity

The anti-bacterial bioactivity profile of the synthesized derivatives was performed by broth microdilution method using tetrazolium microplate assay (TEMA) [19]. All the hybrid molecules were screened in vitro against two Gram-positive bacteria (*S. pneumoniae* and *S. aureus*) and three Gram-negative bacteria (*E. coli, E. aerogenes* and *S. typhi*) and the MIC was reported in μg/mL. The bacterial cultures were freshly grown, emulsified in Muller Hinton broth (MHB) and incubated until the log phase growth was achieved. Its turbidity was then matched to McFarland standard no. 0.5 to achieve the inoculum concentration of 1.5 × 10^8^ CFU/mL. The test was performed in triplicates making serial twofold concentrations ranging between 3.91 and 250 μg/mL. Coloring reagent 3-(4,5-dimethyl-2-thiazolyl)-2,5-diphenyl-2*H*-tetrazolium bromide (MTT) was used to identify the results. The MIC was calculated as the lowest concentration of compounds that prevented the colour change from yellow to purple. DMSO was used as a negative control in this assay while streptomycin, kanamycin and vancomycin were used as positive controls.

#### In-vitro evaluation of anti-tuberculosis activity

A well-characterized H37Rv ATCC 25618 virulent strain of *M. tuberculosis* was used to complete the anti-tuberculosis activity of the synthesized compounds by colorimetric microdilution assay, using tetrazolium salt as a colouring reagent, following our previously reported broth micro dilution method and by using isoniazid as a positive control and DMSO as a negative control [19]. The mycobacterial inoculum was prepared by a 5 day old freshly grown culture in Middlebrook 7H9 broth, supplemented with 0.2% glycerol, 0.05% Tween 80 and 10% albumin, dextrose and catalase (ADC) supplement. The inoculum turbidity was adjusted to McFarland standard no. 1 to achieve the concentration of 3 × 108 CFU/mL. Middlebrook 7H9 broth supplemented with oleic, albumin, dextrose and catalase (OADC) was then used to further dilute it, in a ratio of 1:20. 2-Fold serial dilution was made in 96-well microtiter plate in the range of 0.195–50 μg/mL. Each microtiter plate was sealed and incubated for 5 days at 37 °C in 8% CO_2_, followed by the addition of 50 μL of tetrazolium-tween 80 mixtures (1.5 mL of 1 mg/mL MTT in absolute ethanol and 1.5 mL of 10% Tween-80). After tetrazolium addition, the plates were incubated again for the next 24 h at 37 °C. Next day the bacterial viability was registered for each well based on the color change of yellow MTT to purple formazan and the MIC was defined as the lowest concentration of compound that totally inhibited bacterial growth (no color change). The assays were performed in triplicates.

#### Protocol of molecular modelling and docking

Molecular docking study for all compounds was performed to predict the anti-dengue activity on structural basis of coumarin derivatives. Binding interactions ability and orientations direction of the most active inhibitors to the potent site of the enzyme pocket were used to predict their binding modes, binding affinities, and orientations at the active site of the enzyme, A 3D structure of the enzyme was derived from Protein Data Bank website with code (PDB ID: 2FOM). All water molecules and hetero groups were removed from the receptor crystal structure beyond the radius of 5 Å of the reference ligands and protein structure was refined by employs OPLS-2005 force field calculations and minimization using the Protein Preparation Wizard™ software. The Receptor Grid Generation™ applied to generate active sites residues and used it to dock the optimized ligands into the respective receptor. The structures of all compounds were drawn using ChemDraw Ultra from the ChemOffice software package. Then, it was imported into ligands preparation and optimization by using LigPrep™ application were performed with OPLS-2005 force field calculation also to generate the lowest energy state of each ligand. Docking binding stimulation was finally carried out for five poses per ligand and the pose with highest score was displayed and recorded for each ligand [35–37].

## Conclusions

In the present work, conjugated thiazolidinone molecules (**SKYa**–**SKYg**) derived from coumarins linked by hydrazine moiety have been successfully synthesized by application of Pearson’s HSAB principle. Anti-bacterial and anti-TB activity testing of all the molecules revealed that most of the hybrids displayed activity against the bacterial and tubercle cells. In particular, compound **SKYb** exhibited the highest anti-bacterial profile against all the pathogens. Significantly, the analogue **SKYc**, **SKYd** and **SKYe** also displayed potent activities (99–378 μg/mL). Compound **SKYa** displayed enhanced anti-TB activity. Results also showed considerable anti-TB activity by compound **SKYb** (MIC 132 μg/mL). Importantly, anti-dengue results concluded that conjugate **SKYf** exhibited the most potent activity (DS − 4.014) followed by compound **SKYd** (DS − 3.964), compound **SKYc** (DS − 3.905) and compound **SKYe** (DS − 3.889). Compounds **SKYg** (DS − 2.992), **SKYb** (DS − 2.960) and **SKYa** (DS − 2.754) also displayed very good results when all were compared to the standards 4-hydroxypanduratin (DS − 3.379), panduratin (DS − 3.189) and ethyl 3-(4-(hydroxymethyl)-2-methoxy-5-nitrophenoxy)propanoate (DS –3.381). Docking results proved that the hydrophobic interaction between compounds and protein, inside the active pocket is the most important interaction to increase the activity of compounds against the dengue virus. This study presents novel 4-thiazolidinone-coumarin-hydrazine hybrids as potential lead molecules for further structural optimization as anti-bacterial, anti-TB and anti-dengue agents.

## Additional file

**Additional file 1.** Additional figures and Tables.
