# Supplementary material for: 4-Thiazolidinone coumarin derivatives as two-component NS2B/NS3 DENV flavivirus serine protease inhibitors: synthesis, molecular docking, biological evaluation and structure–activity relationship studies
Source: Chem Cent J. 2018 Jun 12;12:69. doi: 10.1186/s13065-018-0435-0 (PMC5997609; doi:10.1186/s13065-018-0435-0)
Supplement: Supplementary file 1 — Additional file 1. Additional figures and Tables. [file 13065_2018_435_MOESM1_ESM.pdf]

Table S1: Percentage yields, melting points, CHN and IR spectral data of the fourth series of thiazolidinone coumarin derivatives (**SKYa-SKYg**).

Compound	yield (%)	Mp (°C)	CHN (%)	IR (ν, cm^{-1})			
				NH	OH	C=O keto	S-C=N
**SKYa**	63.1	256-258	C, 55.86; H, 3.64; N,13.90	3156.15	-	1626.05	1609.07
**SKYb**	67.6	249-251	C, 44.18; H, 2.69; N, 11.0	3152.26	-	1690.11	1622.09
**SKYc**	78.2	261-263	C, 53.03; H, 3.53; N, 13.28	3096.74	3450.20	1693.97	1625.15
**SKYd**	76.7	248-250	C, 54.41; H, 3.91; N, 12.64	3143.76	-	1702.14	1621.37
**SKYe**	79.1	259-261	C, 54.33; H, 4.0; N, 12.62	3120.09	-	1715.02	1612.77
**SKYf**	81.6	269-271	C, 54.32; H, 4.1; N, 12.60	3233.60	-	1684.00	1608.48
**SKYg**	78.9	235-237	C, 48.85; H, 2.87; N, 16.22	3134.72	-	1683.40	1622.59

Table S2: ^{1}H &^{13}C NMR signals and LC-MS data of the fourth series of thiazolidinone coumarin derivatives (**SKYa-SKYg**)

Compound	Chemical shift ^{1}H NMR ($\delta_H$ppm)			Chemical shift ^{13}C NMR (δ_C ppm)				LCMS (+ESI)
	NH	Coumarin H-4	Coumarin H-5	Thiazolidinone CH_2	Coumarin C-4	Coumarin C-5	Thiazolidinone CH_2	Thiazolidinone S-C-NH	m/z (amu)
**SKYa**	12.24	8.19	7.87	3.91	141.53	129.28	32.85	173.90	302.0578
**SKYb**	12.23	8.16	8.16	3.90	140.14	131.22	32.95	174.17	381.0902
**SKYc**	11.71	8.10	7.69	3.88	142.11	129.26	32.80	173.91	318.2988
**SKYd**	12.10	8.13	7.44	3.88	155.54	130.45	32.83	173.93	332.0697
**SKYe**	12.00	8.12	7.79	3.87	155.54	130.45	32.83	173.93	332.0705
**SKYf**	12.01	8.16	7.41	3.89	142.84	124.70	32.85	173.88	332.0696
**SKYg**	12.21	8.11	7.39	3.92	140.14	131.20	35.89	173.12	347.0403

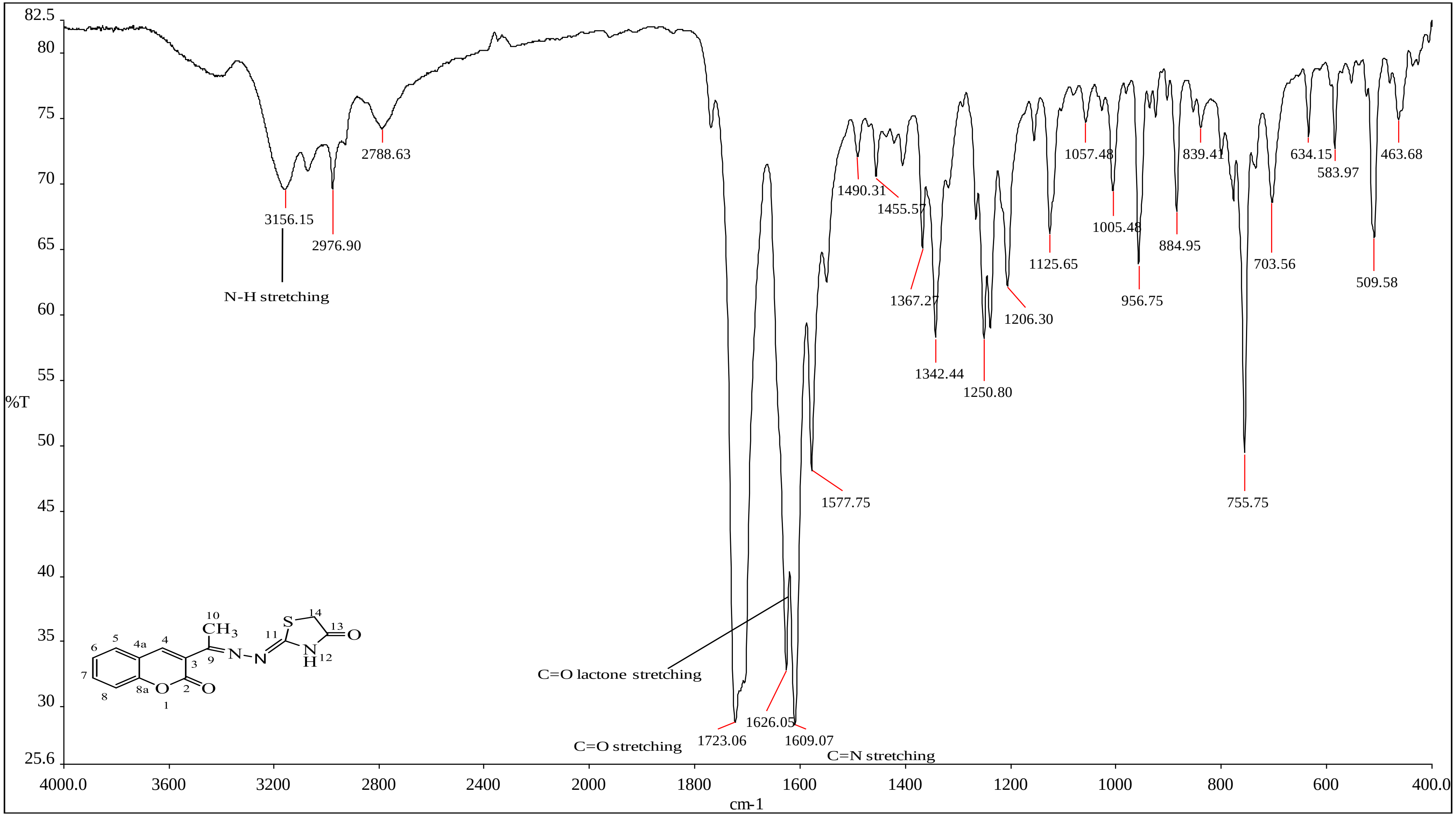

Figure S1: IR spectrum of **SKYa** (KBr)

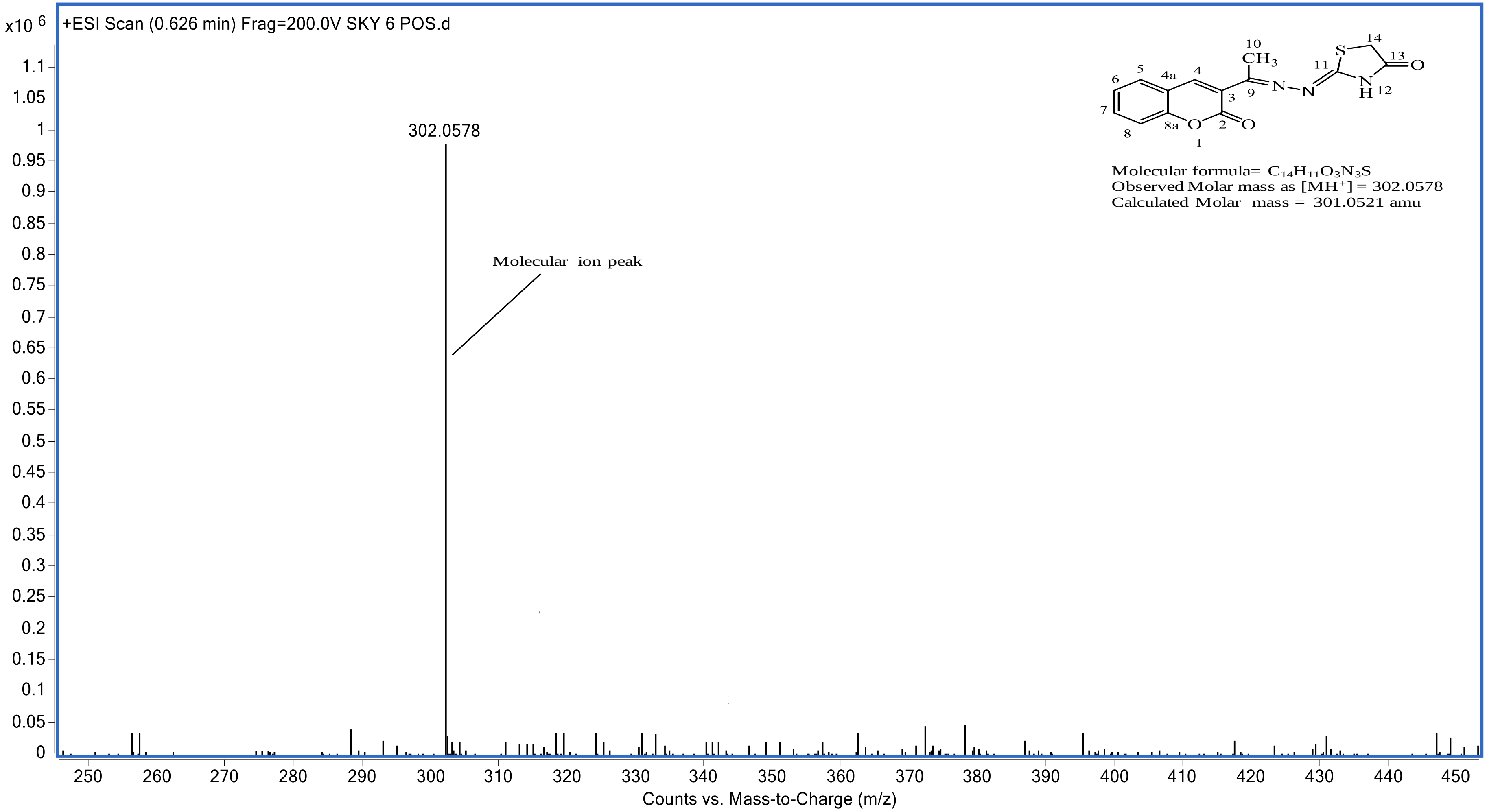

Figure S2: LC-MS spectrum of **SKYa** (+ESI)

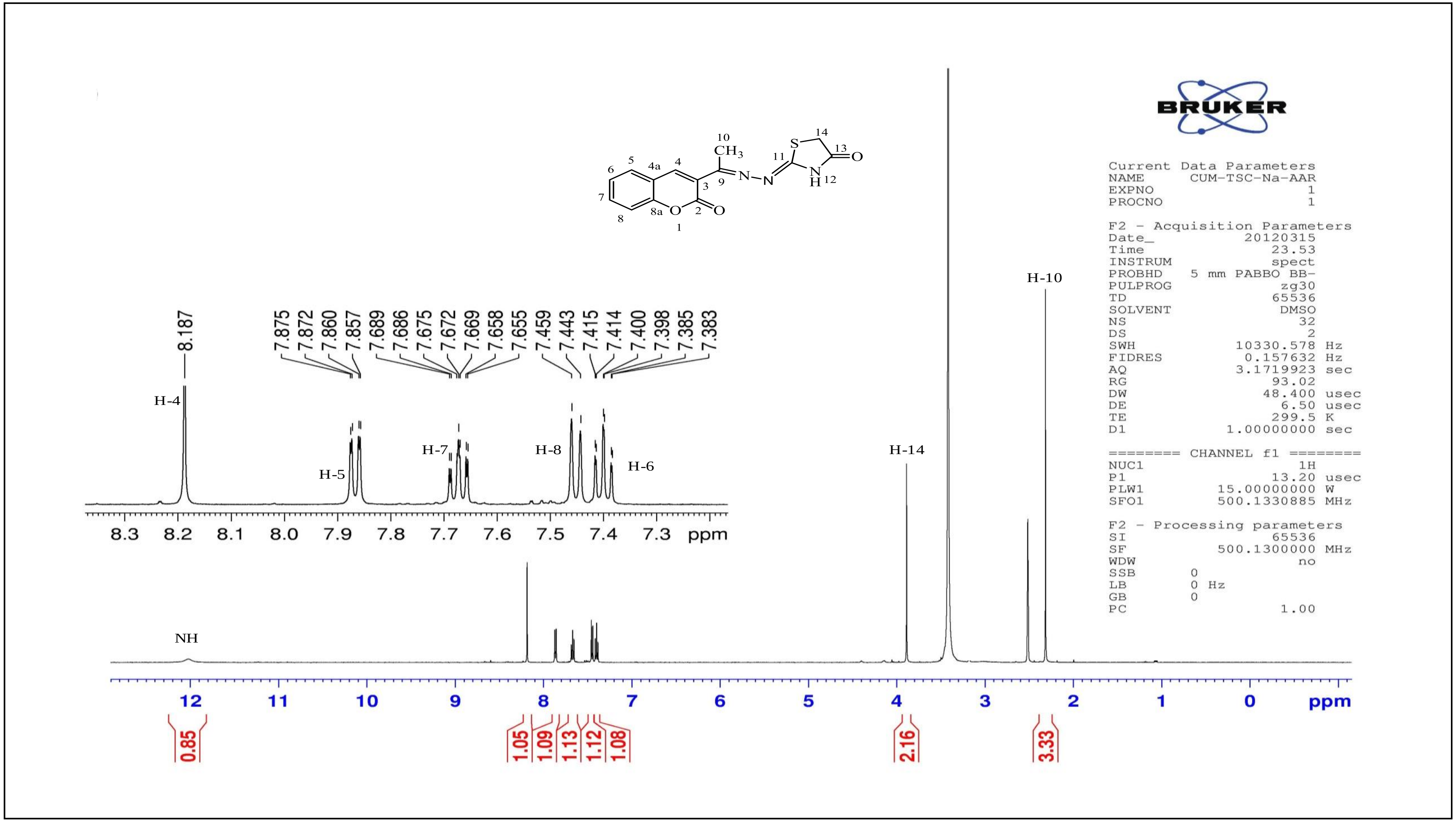

Figure S3: ^{1}H NMR spectrum of **SKYa** (500MHz) in DMSO-d_6

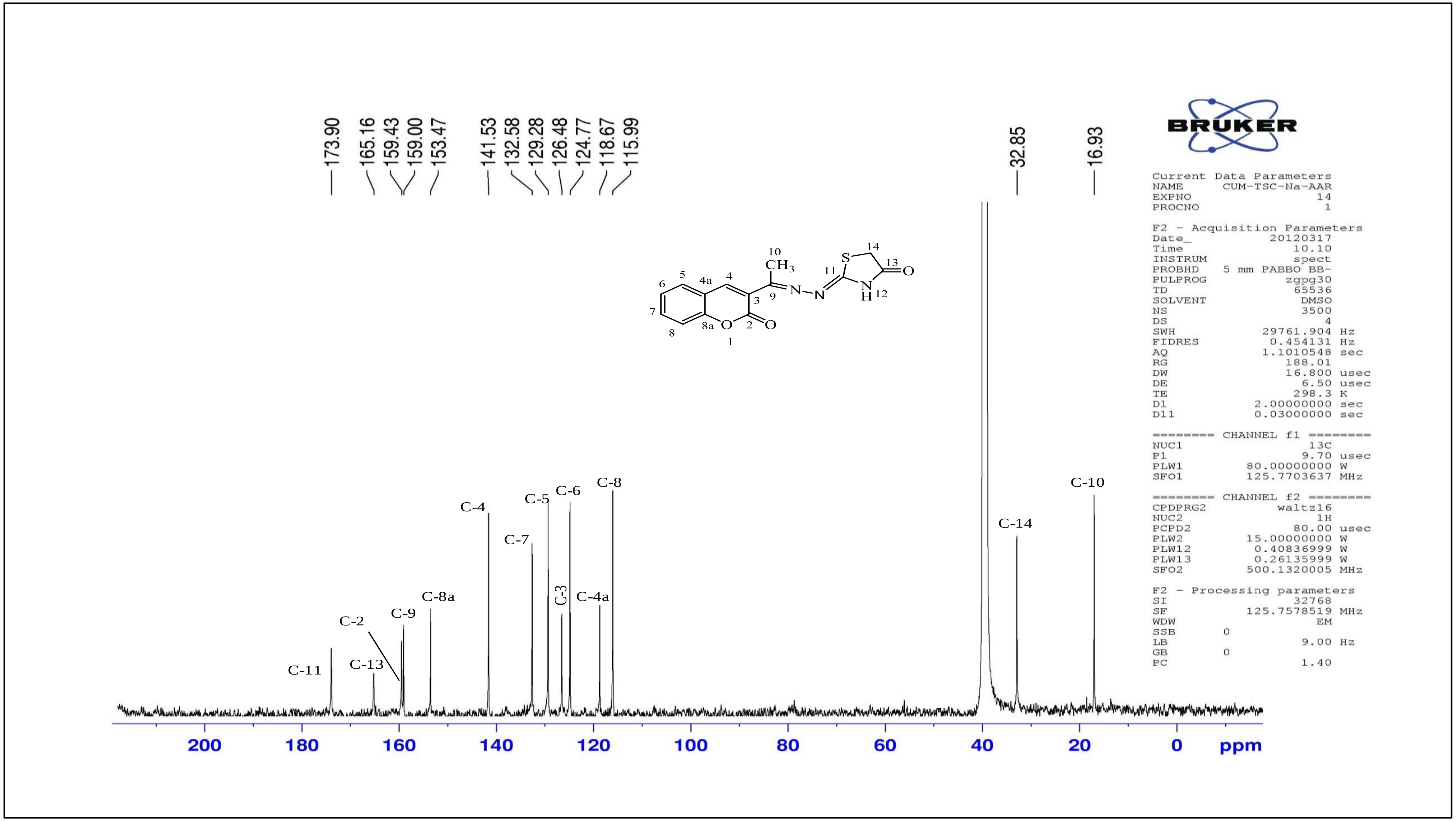

Figure S4: ^{13}C NMR spectrum of **SKYa** (125MHz) in DMSO-d_6

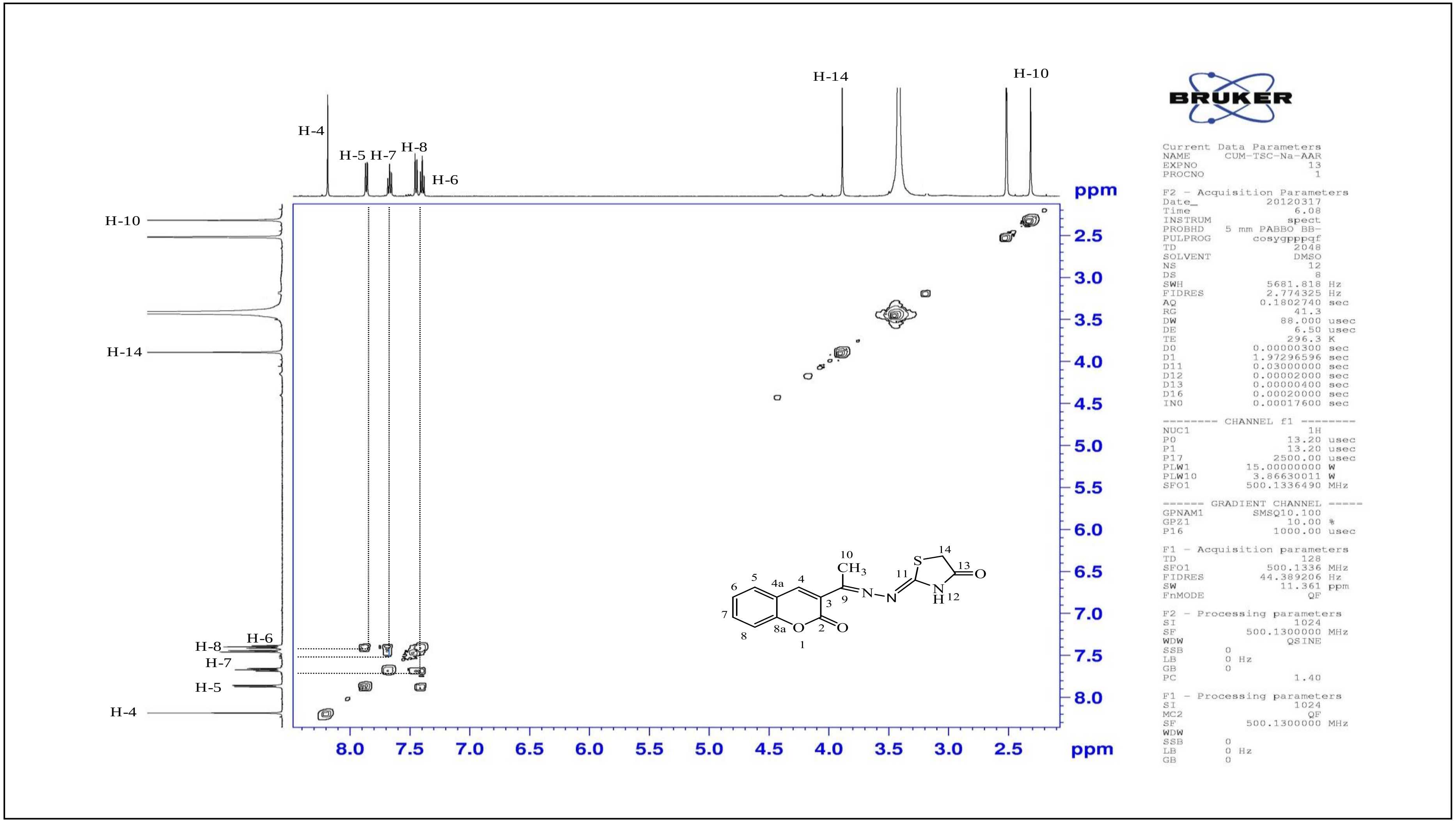

Figure S5: ^{1}H-^{1}H COSY spectrum of **SKYa** (500MHz) in DMSO-d_6

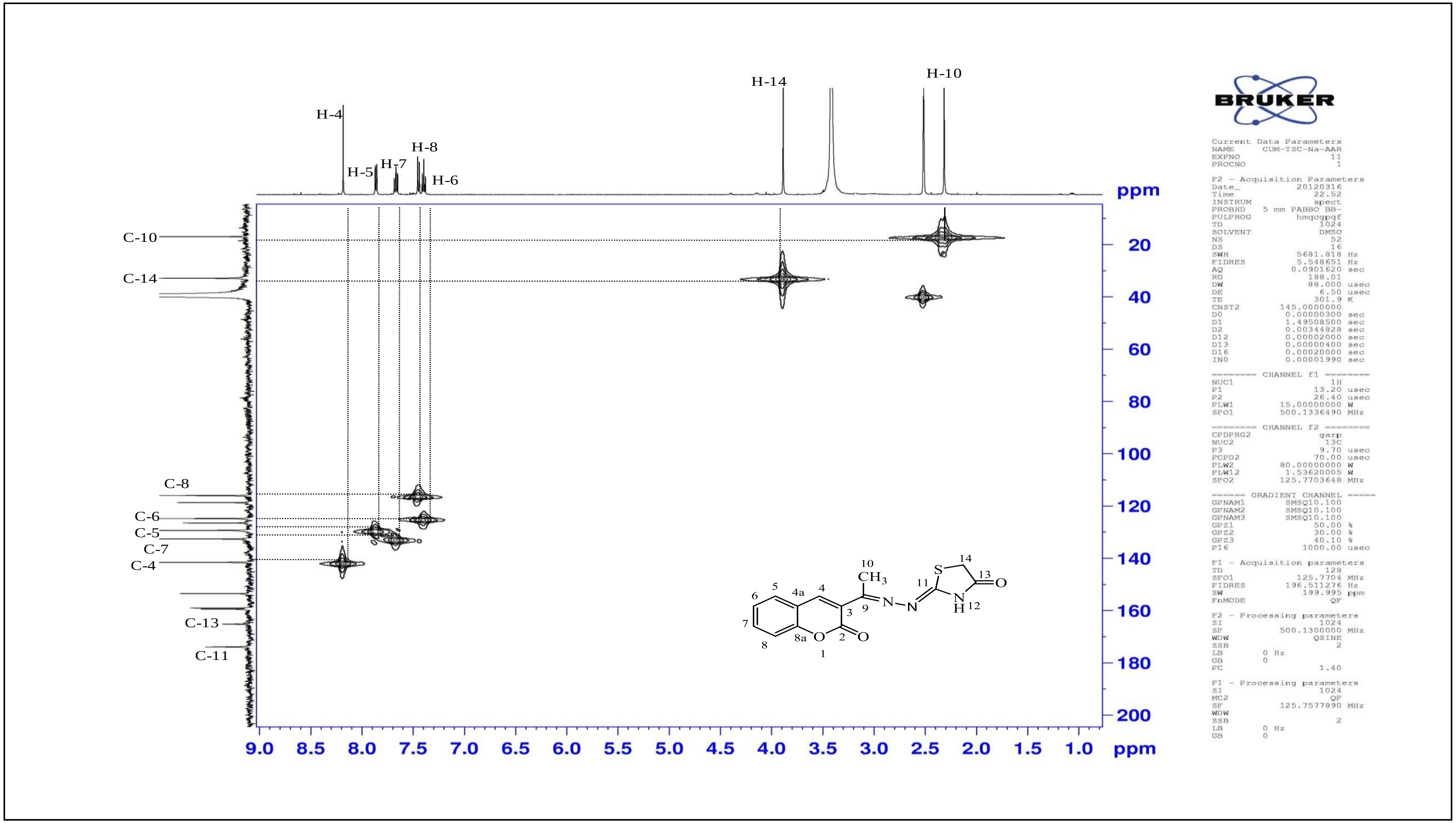

Figure S6: ^{1}H-^{13}C HMQC spectrum of **SKYa** (500MHz) in DMSO-d_6

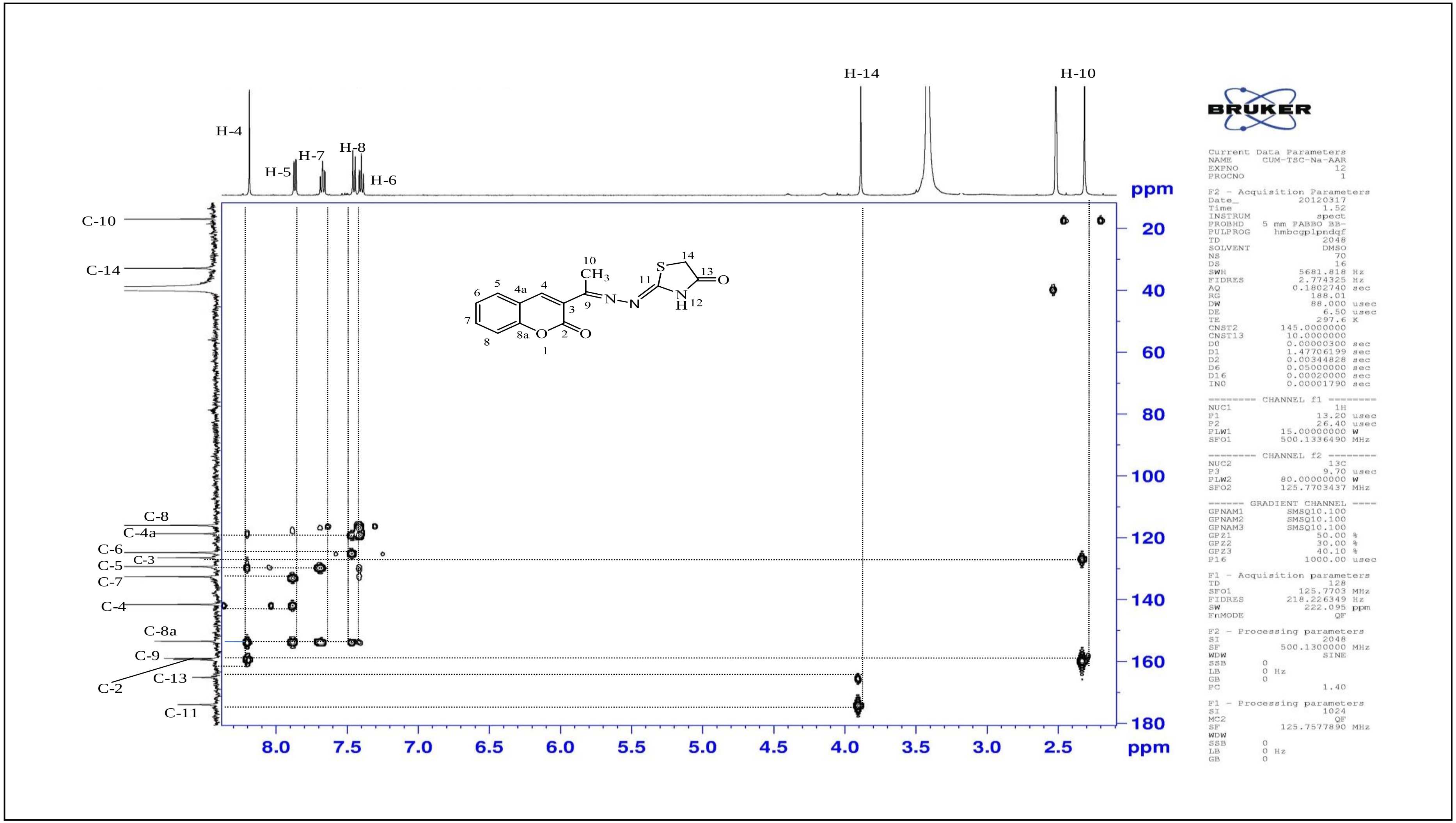

Figure S7: ^{1}H-^{13}C HMBC spectrum of **SKYa** (500MHz) in DMSO-d_6

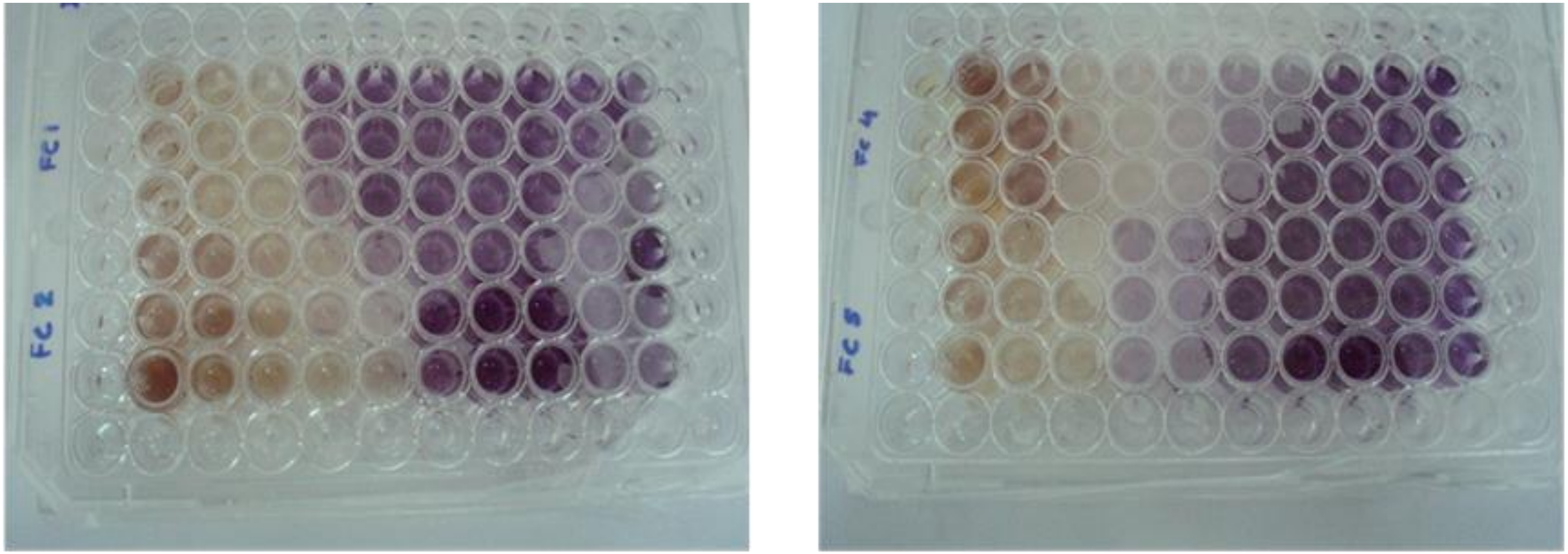

Figure S8: Microtiter plates displaying results.
